# Adipocyte-specific ablation of the Ca^2+^ pump SERCA2 impairs whole-body metabolic function and reveals the diverse metabolic flexibility of white and brown adipose tissue

**DOI:** 10.1016/j.molmet.2022.101535

**Published:** 2022-06-24

**Authors:** Marco Bauzá-Thorbrügge, Elin Banke, Belén Chanclón, Eduard Peris, Yanling Wu, Saliha Musovic, Cecilia Jönsson, Peter Strålfors, Patrik Rorsman, Charlotta S. Olofsson, Ingrid Wernstedt Asterholm

**Affiliations:** 1 Department of Physiology/Metabolic Physiology, Institute of Neuroscience and Physiology, The Sahlgrenska Academy at University of Gothenburg, Medicinaregatan 11, SE-405 30 Göteborg, Sweden; 2 Department of Biomedical and Clinical Sciences, Linköping University, SE-58185 Linköping, Sweden; 3 Oxford Centre for Diabetes, Endocrinology and Metabolism, Radcliffe Department of Medicine, University of Oxford, Oxford OX4 7LE, UK

**Keywords:** Obesity, Adipose tissue, Type-2 diabetes, Endoplasmic reticulum, Adipokine, Brown adipose tissue, Calcium, SERCA2, Mitochondria, FGF21

## Abstract

**Objective:**

Sarco/endoplasmic reticulum Ca^2+^-ATPase (SERCA) transports Ca^2+^ from the cytosol into the endoplasmic retitculum (ER) and is essential for appropriate regulation of intracellular Ca^2+^ homeostasis. The objective of this study was to test the hypothesis that SERCA pumps are involved in the regulation of white adipocyte hormone secretion and other aspects of adipose tissue function and that this control is disturbed in obesity-induced type-2 diabetes.

**Methods:**

SERCA expression was measured in isolated human and mouse adipocytes as well as in whole mouse adipose tissue by Western blot and RT-qPCR. To test the significance of SERCA2 in adipocyte functionality and whole-body metabolism, we generated adipocyte-specific SERCA2 knockout mice. The mice were metabolically phenotyped by glucose tolerance and tracer studies, histological analyses, measurements of glucose-stimulated insulin release in isolated islets, and gene/protein expression analyses. We also tested the effect of pharmacological SERCA inhibition and genetic SERCA2 ablation in cultured adipocytes. Intracellular and mitochondrial Ca^2+^ levels were recorded with dual-wavelength ratio imaging and mitochondrial function was assessed by Seahorse technology.

**Results:**

We demonstrate that SERCA2 is downregulated in white adipocytes from patients with obesity and type-2 diabetes as well as in adipocytes from diet-induced obese mice. SERCA2-ablated adipocytes display disturbed Ca^2+^ homeostasis associated with upregulated ER stress markers and impaired hormone release. These adipocyte alterations are linked to mild lipodystrophy, reduced adiponectin levels, and impaired glucose tolerance. Interestingly, adipocyte-specific SERCA2 ablation leads to increased glucose uptake in white adipose tissue while the glucose uptake is reduced in brown adipose tissue. This dichotomous effect on glucose uptake is due to differently regulated mitochondrial function. In white adipocytes, SERCA2 deficiency triggers an adaptive increase in fibroblast growth factor 21 (FGF21), increased mitochondrial uncoupling protein 1 (UCP1) levels, and increased oxygen consumption rate (OCR). In contrast, brown SERCA2 null adipocytes display reduced OCR despite increased mitochondrial content and UCP1 levels compared to wild type controls.

**Conclusions:**

Our data suggest causal links between reduced white adipocyte SERCA2 levels, deranged adipocyte Ca^2+^ homeostasis, adipose tissue dysfunction and type-2 diabetes.

## Highlights

• Adipocyte SERCA2 is downregulated in human subjects with type-2 diabetes.
• Loss of SERCA2 disturbs the intracellular Ca^2+^ homeostasis in adipocytes.
• Impaired metabolism and altered adipokine levels in adipocyte-SERCA2 null mice.
• Loss of SERCA2 accelerates metabolic processes in white adipocytes.
• Loss of SERCA2 impairs the mitochondrial function of brown adipocytes.

## Graphical abstract

**Figure:**
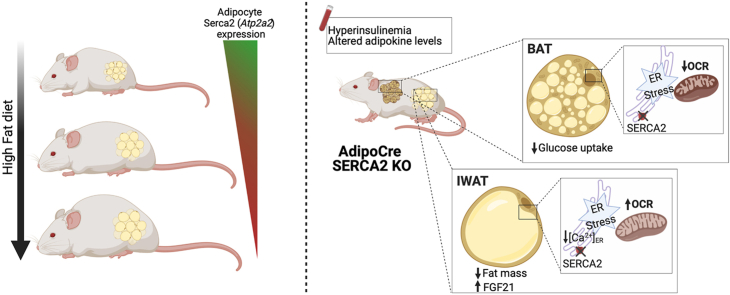

## 1 Introduction

The white adipose tissue (WAT) is important for maintenance of metabolic homeostasis in the body. Besides storing excess energy in the form of triglycerides, WAT also regulates whole-body metabolism by secreting a large variety of bioactive molecules, referred to as adipokines. Adipokines may be cytokines (e.g. interleukins and tumour necrosis factor alpha) whereas others, including adiponectin, leptin and resistin, are protein hormones. Obese adipose tissue is commonly dysfunctional with decreased triglyceride storage capacity and disturbed adipokine release, leading to ectopic lipid deposition, chronic low-grade inflammation, and systemic insulin resistance [1,2].

The endoplasmic reticulum (ER) constitutes the folding compartment for proteins entering a secretory pathway and is also a Ca^2+^ reservoir, important for controlling the dynamic regulation of Ca^2+^-dependent cellular functions including proliferation and differentiation, contraction, gene transcription, hormone secretion and apoptosis. Disrupted ER function causes mis- or unfolded proteins to accumulate within the ER and activates a process known as unfolded protein response (UPR). A high concentration of Ca^2+^ within the ER is essential for the normal function of chaperones and enzymes involved in protein folding and thus for the ability of UPR to resolve ER stress [3]. Moreover, intracellular Ca^2+^ has been shown to regulate several adipocyte-specific processes, such as lipolysis [[4], [5], [6]] and glucose uptake [7,8]. Ca^2+^ is also important for regulation of the secretion of adipocyte hormones, such as the appetite-controlling hormone leptin [[9], [10], [11]]. In earlier studies, we defined an important regulatory role of Ca^2+^ for secretion of the adipocyte-specific insulin-sensitizing hormone adiponectin; Ca^2+^ potently augments cAMP-stimulated adiponectin exocytosis and is also required for sustained adiponectin release over prolonged time periods [12,13].

The regulation of Ca^2+^ fluxes between ER and the cytosol is largely dependent on sarco/endoplasmic reticulum Ca^2+^ ATPase (SERCA) pumps that transport Ca^2+^ from the cytosol into the ER. There are three paralogous genes that code for SERCA: *Serca1* (*Atp2a1*), *Serca2* (*Atp2a2*), and *Serca3* (*Atp2a3*). SERCA activity has also been linked to uptake of Ca^2+^ in the mitochondria, another important intracellular Ca^2+^ storage compartment [14]. The sequestration of Ca^2+^ within mitochondria impacts oxidative phosphorylation as well as cytosolic Ca^2+^ signals and excessive mitochondrial Ca^2+^ can trigger cell death (as reviewed in [15,16]). Interestingly, studies in the heart, the liver and in pancreatic insulin-secreting beta-cells have affirmed SERCA pumps as key players in the pathophysiology of type-2 diabetes (T2D) [[17], [18], [19], [20], [21]]. However, diseases associated with disrupted ER Ca^2+^ dynamics have received insufficient attention and the role of SERCA for adipose tissue functionality is largely uninvestigated.

Here we show that SERCA2 is the paralog chiefly expressed in white mouse adipocytes isolated from both subcutaneous and visceral depots. Our study also demonstrates that SERCA2 is downregulated in adipocytes isolated from high fat diet (HFD)-induced obese mice and from human subjects with obesity and T2D. Moreover, genetic ablation of this Ca^2+^ pump results in a disturbed secretion of adipocyte hormones and impaired mitochondrial function in white and brown adipocytes, associated with whole-body glucose intolerance. Our study highlights an important causal link between a disturbed Ca^2+^ homeostasis in adipocytes and metabolic disease.

## 2 Material and methods

### 2.1 Reagents and tools table

**Table:** 

Reagent	Source	Identifier
Antibodies		
Anti-Hsp60 antibody	Abcam	Ab46798
Total OXPHOS Rodent WB Antibody Cocktail	Abcam	Ab110413
Goat anti-mouse IgG	Abcam	Ab6788
Anti-UCP1 antibody	Abcam	Ab10983
Anti-SERCA2 ATPase antibody	Thermo Fisher	MA3-919
Anti- Beta-Tubulin antibody	Cell Signaling	2128
Anti-Cleaved Caspase 3 antibody	Cell Signaling	9661
Anti-GAPDH antibody	Abcam	Ab8245

Chemicals, peptides, and recombinant proteins		
QIAzol	Qiagen	79306
Fast SYBR Green Master Mix	Thermo Scientific	4385617
(±)-Norepinephrine (+)-bitartrate salt (NE)	Sigma Aldrich	A0937
Tamoxifen	Sigma Aldrich	T5648
Bovine serum albumin	Sigma Aldrich	A7030
DMEM, high glucose	Life Technology	41965-039
Seahorse XF DMEM Medium, pH 7.4	Agilent	103575-100
Penicillin/streptomycin	Thermo Scientific	15140-122
Seahorse XF Glucose (1.0 M solution)	Agilent	103577-100
Seahorse XF Pyruvate (100 mM solution)	Agilent	103578-100
Seahorse XF l-Glutamine (200 mM solution)	Agilent	103579-100
Dexamethasone	Sigma Aldrich	D4902
Rosiglitazone	Sigma Aldrich	R2408
Insulin Actrapid® Penfill®	Novo Nordisk	A10AB01
3-Isobutyl-1-methylxanthine (IBMX)	Sigma Aldrich	I5879
DMSO	Sigma Aldrich	D5879
PhosSTOP	Sigma Aldrich	4906845001
Hoechst	Thermo Scientific	H-21486
CM-H2DCFDA	Thermo Scientific	C6827
Mitotracker Green FM	Thermo Scientific	M7514
Ultima Gold scintillation cocktail	Perkin Elmer	6013326
D-[U-^14^C]-glucose (5 μCi/mouse)	Perkin Elmer	NEC042x25OUC
Diisononyl phthalate	Sigma–Aldrich	376663
Fura-2 AM (Life Technologies)	Thermo Scientific	F1221
Fetal Bovine Serum (FBS Hyclone)	Gibco	10309433
New Born Calf Serum (NBCS)	Gibco	11530636
RU360	Sigma Aldrich	557440
Cyclopiazonic acid	Sigma Aldrich	C1530
Forskolin	Sigma Aldrich	F6886
CL 316243 disodium salt	Tocris	1499
ATP magnesium salt	Sigma Aldrich	A9187
Thapsigargin	Tocris	1138
Eosin	Sigma Aldrich	HT110116
Hematoxylin	Sigma Aldrich	GHS316
*N*,*N*,*N*′,*N*′-Tetramethyl-*p*-phenylenediamine (TMPD)	Sigma Aldrich	T7394
Trans-Blot® Turbo™ Midi PVDF Transfer Packs	Bio-Rad	1704157
10× Tris/glycine/sds	Bio-Rad	161-0772
l-Ascorbic acid	Sigma Aldrich	A0278
Succinate	Sigma Aldrich	S2378
Antimicyn A	Sigma Aldrich	A8674
Rotenone	Sigma Aldrich	R8875
d-(+)-Malic acid	Sigma Aldrich	02300
pluriStrainer® (100 μm)	Pluriselect	43-50100-51
Rhod-2, AM	Thermo Scientific	R1245MP
Sodium Palmitate	Sigma Aldrich	P9767
Critical commercial assays		
BCA protein assay kit	Thermo Scientific	CAT#23225
Seahorse XFe96 FluxPax	Agilent	CAT#102416-100
High-Capacity cDNA Reverse Transcription Kit	Thermo Scientific	CAT#4368814
Bradford	Amresco	M172-1L
Mouse Insulin ELISA kit	Mercodia	10-1247-01,
Total adiponectin ELISA kit	EMD Millipore	EZMADP-60K
High molecular weight (HMW) adiponectin ELISA kit	MyBiosource	MBS028367
Leptin DuoSet ELISA kit	R&D Systems	DY498
Resistin DuoSet ELISA kit	R&D Systems	DY1069
Total adiponectin DuoSet ELISA kit	R&D Systems	DY1119
ReliaPrep™ RNA Cell Miniprep System	Promega	Z6112
Seahorse XF Cell Mito Stress Test Kit	Agilent	103015-100
MouseWG-6 v2.0 Gene Expression BeadChips	Illumina	NA
Triglyceride kit	Randox	TR210
Mouse FGF-21 ELISA Kit	Abcam	ab212160


Experimental models: Cell lines		
3T33T3-L1 preadipocytes	Zenbio	SP-L1-F


Experimental models: Organisms/Strains		
LoxP flanked SERCA2 knock-in *C57B6* mice	Geir Christensen (UiO, Norway)	N/A
Mouse Adipoq-Cre (B6; FVB-Tg(Adipoq-cre)1Evdr)	Jackson Laboratory	J stock 010803
Mouse Adipoq-CreER (B6.129-Tg(Adipoq-cre/Esr1∗)1Evdr)	Jackson Laboratory	J Stock 024671


Oligonucleotides		
RT qPCR Primers	This Paper	Table S1


Software and Algorithms		
Graphpad Prism 7.0 for statistical analysis	GraphPad	N/A
Image Lab	Bio-Rad	N/A

### 2.2 Experimental model and subject details

#### 2.2.1 Genetically modified mouse lines

C57BL/6 background (Jackson Laboratory) loxP flanked SERCA2 knock-in mice were obtained from Geir Christensen (UiO, Oslo, Norway) and crossed with adiponectin promoter driven Cre transgenic mice with C57BL/6 background (B6; FVB-Tg (Adipoq-cre)1Evdr/J stock 010803, Jackson Laboratory) to selectively knock-out SERCA2 from adipocytes. Littermates not expressing Cre were used as wildtype controls. Male or female 8–9-week-old C57BL/6 mice (Jackson Laboratory) were fed chow (Global Diet #2016, Harlan-Teklad) or high fat diet (HFD, 60% kcal from fat: D12492, Research Diets Inc.) through 8 weeks. To obtain enough primary SERCA2 knockout adipocytes for *ex vivo* adipocyte hormone secretion experiments, SERCA2-loxP mice were crossed into CreER and adiponectin promoter-driven transgenic mice (Jackson Laboratory, B6.129-Tg (Adipoq-cre/Esr1∗)1Evdr/J Stock 024671) to generate an inducible adipocyte-specific SERCA2 knockout mouse model. Tamoxifen (Sigma–Aldrich T5648) was dissolved in sunflower oil and given by gavage (5 mg/mouse) at 10 weeks. After two weeks, the mice were euthanized, and adipose tissue was dissected. All animal work was approved by the Regional Ethical Review Board in Gothenburg Sweden. All mice are male unless otherwise specified in the text, graph, or legend.

#### 2.2.2 Mouse adipocyte cultures

3T3-L1 preadipocytes (ZenBio) cells were maintained as sub confluent cultures in DMEM (high-glucose, 4500 mg; Life Technologies, Stockholm, Sweden) containing 10% fetal bovine serum (Thermo Fisher Scientific, Waltham, MA, USA) and 1% penicillin–streptomycin (medium 1; Life Technologies) and differentiated into mature adipocytes as previously described [13]. Briefly, cells were grown to confluence (day 0) and thereafter incubated in medium 1 supplemented with 1 μM dexamethasone, 850 nM insulin, and 0.5 mM 3-isobutyl-1-methylxanthine (IBMX). After 48 h (day 2) the medium was changed to medium 1 supplemented with insulin only. After an additional 48 h (day 4) the medium was then replaced by medium 1 alone and was thereafter freshly replaced every 2 days. Experimental studies were performed in mature 3T3-L1 adipocytes between days 8 and 10 from the start of differentiation.

IWAT stromal vascular fraction (SVF) were isolated as previously described [22]. IWAT SVF were differentiated into mature adipocytes following the same protocol used for 3T3-L1 preadipocyte, but with the addition of 1 μM rosiglitazone (Sigma) in the first differentiation step. Brown fat SVF was isolated and differentiated as previously described [23]. The cell culture medium was DMEM, supplemented with 10% new-born calf serum (Thermo Fisher Scientific, Waltham, MA, USA), 4 nM insulin, 4 mM glutamine, antibiotics (50 IU penicillin/mL and 50 μg streptomycin/mL), 10 mM HEPES, and 25 μg/mL sodium ascorbate (Sigma–Aldrich, St. Louis, MO, USA). The medium was changed on days 1, 3, 6 and 9. The SVF IWAT and BAT adipocytes cells were typically used for experiment at day 10 when they were fully differentiated as judged by their lipid content. For hypoxia treatment, adipocytes were cultured in 5% O_2_ in a InvivO_2_ 300 hypoxia workstation (Baker Ruskinn, Bridgend, United Kingdom) at 37 °C. Normoxia control cells remained to be cultured in the humidified atmosphere (5% CO_2_, 95% air) at a temperature of 37 °C. For Palmitate experiments, cells were treated with 250–500 μM palmitate conjugated with fatty acid-free bovine serum albumin (BSA) for 24 h. The stock solution was prepared by conjugating palmitate with BSA following Seahorse Bioscience protocol (Agilent, USA). Briefly, palmitate was dissolved in 150 mM sodium hydroxide at 70 °C and mixed with 0.17 mM fatty acid-free BSA at 50 °C for 30 min, yielding a final stock solution of 1 mM. The molar ratio of FFA/BSA was 6:1.

#### 2.2.3 Human subjects

Informed consent was obtained from all participants after oral and written information. The procedures have been approved by the Regional Ethical Board, Linköping University, and were performed in accordance with the WMA Declaration of Helsinki. Adult women who were undergoing elective gynaecological abdominal surgery under general anaesthesia at the Department of Obstetrics and Gynaecology at the University Hospital in Linköping were recruited consecutively. A slice of abdominal subcutaneous adipose tissue from skin to muscle fascia was excised, and adipocytes were immediately isolated.

### 2.3 Experimental procedures

#### 2.3.1 Isolation of human adipocytes and sample preparation

Human adipocytes were isolated from adipose tissue samples by collagenase (type 1, Worthington, NJ, USA) as described previously [24]. Adipocytes were lysed after separating cells from medium by centrifugation through dinonylphtalate oil. To minimize modification of proteins cells were immediately dissolved in SDS with protease and protein phosphatase inhibitors, frozen within 10 s, and later thawed in boiling water for further processing for SDS-PAGE, as described [25].

#### 2.3.2 Oral glucose tolerance test

Glucose (2.5 mg/g mouse) was given by oral gavage after a 4 h fast and blood samples were collected at 0, 15, 30, 60 and 120 min. Adipose tissue and liver were dissected for further analysis with immunohistochemistry, RT-qPCR or western blotting as indicated.

#### 2.3.3 Glucose uptake

An oral load of D-[U-^14^C]-glucose (5 μCi/mouse, Perkin Elmer, Boston, MA, USA) in 1.39 M of glucose-PBS solution was given to adult mice by gavage after 4 h fasting. After 2 h, tissue was weighed and collected in a 2:1 chlorofom:methanol solution, homogenized in a tissue lyser and stored at 4 °C overnight. CaCl_2_ 1 M was added to all samples and centrifuged at 4 °C 3000 rpm during 20 min to separate aqueous and organic phases. The incorporation of ^14^C was quantified in both phases using Ultima Gold scintillation cocktail (Perkin Elmer, Boston, MA, USA) and a beta counter (Perkin Elmer, Boston, MA, USA). Results are expressed as % of total ^14^C counts per mg tissue.

#### 2.3.4 Measurements of insulin, FGF21 and adipokines

Blood was collected and serum separated by clotting and centrifugation. Serum insulin was measured using a Mouse Insulin ELISA kit (10-1247-01, Mercodia), total adiponectin by the ELISA kit EZMADP-60K (EMD Millipore), high molecular weight (HMW) adiponectin by the ELISA kit MBS028367 (MyBiosource), leptin by the DuoSet ELISA DY498, resistin by the DuoSet ELISA DY1069 (R&D Systems), serum and tissue FGF21 levels by the FGF-21 ELISA Kit ab212160 (Abcam). Adipokines were measured in tissue lysates obtained as previously described [26].

#### 2.3.5 Tissue triglyceride content

The triglyceride content was measured following instructions of manufacturer (Randox Laboratories, Dublin, United Kingdom). Briefly, frozen tissue (30–100 mg of liver) was homogenized using a tissue lyser (3 min at 25 Hz) in chloroform:methanol (2:1), washed with NaCl 0.9% and evaporated overnight. Lipid samples were resuspended in isopropanol and trigycerides were measured.

#### 2.3.6 Isolation of mouse adipose tissue mitochondria

Mitochondria of IWAT or BAT were isolated by differential centrifugation, based upon [27]. Freshly dissected adipose tissue was minced with scissors in ∼10 volumes of ice cold MSHE + BSA (70 mM sucrose, 210 mM mannitol, 5 mM HEPES, 1 mM EGTA and 0.5% (w/v) fatty acid-free BSA, pH 7.2, 4 °C). The tissue was disrupted using a drill-driven Teflon glass homogenizer with 4–5 strokes. Homogenate was centrifuged at 800 *g* for 10 min at 4 °C, the lipid layer was carefully aspirated, and the remaining supernatant was separated and centrifuged at 8000 *g* for 10 min at 4 °C. The pellet was resuspended in MSHE, and the centrifugation was repeated. The final pellet was resuspended for further analysis of oxygen consumption or western blot. Total protein was determined using Bradford Assay reagent (Bio-Rad).

#### 2.3.7 Pancreatic islets isolation

Pancreata were injected through the portal vein with 3–5 mL cold Hanks' buffer containing 0.95 mg/mL collagenase V (Sigma, USA). The collected pancreata were incubated at 37 °C for 7 min. The released islets were washed with Hanks' buffer (0.1% BSA) 3–5 times and then hand-picked under a dissecting microscope to ensure that the islet preparation was pure. The islets were first cultured for 1 h in RPMI 1640 containing 10% FBS, 100 U/mL Penicillin/Streptomycin, 10 mM glucose. After washing the islets once with KRBH buffer + 0.1% BSA, 10–12 islets of similar size were handpicked in 0.3 mL KRBH buffer (0.1% BSA) containing 1 mM glucose. The islets were then pre-incubated KRBH buffer (0.1% BSA) containing 1 mM glucose for 30 min at 37 °C, followed by 1 h incubation in 0.3 mL KRBH buffer (0.1% BSA) containing (in mM): 1, 6 or 20 glucose, 20 glucose + 0.11 Tolbutamide or 20 glucose + 10 Arginine. Insulin was measured in the collected medium.

#### 2.3.8 Western blot analysis

10 μg protein from isolated mitochondria or 50 μg protein from isolated mouse adipocytes or equal volumes of packed human adipocytes were separated on SDS-PAGE gels and electro transferred onto a PVDF membrane. The membranes were blocked with blocking solution (5% BSA in TBS, pH 7.5, containing 0.1% Tween 20) for 1 h and incubated overnight with the corresponding antibodies. Polyclonal antibodies against OXPHOS, UCP1, SERCA2, Beta-Tubulin, GAPDH, HSP60, or cleaved Caspase 3 were used as primary antibodies. Immunoblots were developed using an enhanced chemiluminescence kit. Bands were visualized with the ChemiDoc XRS system (Bio-Rad, CA, USA) and analysed with the program Image Lab© (Bio-Rad, CA, USA) using volume box analysis and the local background subtraction method.

#### 2.3.9 Gene expression analysis

RNA was extracted and purified from adipose tissue and liver using QIAzol® (Qiagen) and ReliaPrep™ RNA Cell Miniprep System (Promega). RNA was reversely transcribed to cDNA by High-Capacity cDNA Reverse Transcription Kit. Fast SYBR Green Master Mix (Thermo Fisher Scientific, Waltham, MA, USA). 5 μg of the original RNA concentration and primers at a concentration of 500 nM were used in the qRT-PCR and gene expression was normalized against β-actin (*Atcb*) using the relative ΔCt method. Primer sequences are provided in Suppl. Table 1. Serca1-3 (*Atp2a1-3*) adipocyte and SVF mRNA expression data in Suppl. Fig. 1A was obtained by DNA microarray using MouseWG-6 v2.0 Gene Expression BeadChips (Illumina).

#### 2.3.10 Histology

Mouse adipose tissue, liver and pancreata were collected and fixed in 4% paraformaldehyde for 24 h. After paraffin embedding and sectioning (5 μm for adipose tissue, liver, and pancreas), tissues were stained with haematoxylin and eosin (H&E). For each animal, images of three distinct regions within two or three sequential histologic sections stained with H&E were used to determine adipocyte size (diameter) and the frequency of crown-like structures (CLS). All CLS were counted and the average number of CLS per image was calculated.

#### 2.3.11 [Ca^2+^]_i_ imaging

Differentiated 3T3-L1 were kept in an extracellular solution (EC) containing (in mmol/L): 140 NaCl, 3.6 KCl, 2 NaHCO_3_, 0.5 NaH_2_PO_4_, 0.5 MgSO_4_, 5 HEPES (pH 7.4 with NaOH), 2.6 CaCl_2_, and 5 glucose. Cells were loaded with fura-2 AM (Life Technologies) and [Ca^2+^]_i_ values were recorded with dual-wavelength ratio imaging as previously described [28]. Excitation wavelengths were 340 and 380 nm, and emitted light was collected at 510 nm. The absolute [Ca^2+^]_i_ was calculated using Eq. 5 in the study by Grynkiewicz et al. (K_d_ = 224 nmol/L) [29]. For measurements of [Ca^2+^] inside mitochondria ([Ca^2+^]mit), cells were incubated with 5 μM rhod-2/AM and 0.02% pluronic F-127 for 30 min at 37 °C [30]. rhod-2/AM was excited at 543 nm and emission was recorded at 576 nm.

#### 2.3.12 Adiponectin and resistin secretion in primary adipocytes

Adiponectin and resistin secretion were measured in mouse primary adipocytes isolated from inguinal white adipose tissue (30-min incubations at 32 °C). Primary adipocytes were isolated from 8-week-old mice inguinal white adipose tissue. Tissue samples were minced and digested using collagenase type II (1 mg/mL; 45–60 min; 37 °C) and poured through a 100-μm nylon mesh. Floating adipocytes were washed with Krebs–Ringer glucose buffer (1% BSA) and immediately used for experiments. Primary adipocytes were diluted to 25% volume/volume. Secretion was measured in an EC (see [Ca^2+^]_i_ imaging) containing test substances as indicated. Primary adipocytes cells were separated from media by centrifugation in diisononyl phthalate (Sigma–Aldrich) followed by snap freezing in dry ice. Tubes were cut through the oil layer at two points. Cells were lysed in PBS containing SDS (2%) and protease inhibitor (1 tablet/10 mL; PhosSTOP, Sigma Aldrich). EC aliquots and cell homogenates were stored at −80 °C. Secreted adiponectin or resistin (measured with mouse ELISA DuoSets; R&D Systems) was expressed in relation to total protein content (BCA protein assay).

#### 2.3.13 Oxygen consumption rate

For the Mitostress test, Agilent instructions were followed. Briefly, adipocytes differentiated onto XF96 Microplates (Seahorse Bioscience, Agilent Technologies, CA, USA) from IWAT or BAT SVF were washed with assay medium and incubated for 1 h at 37 °C. The oxygen consumption rate (OCR) was determined at four levels: with no additions, and after adding oligomycin (2 μM), carbonyl cyanide 4-(trifluoromethoxy) phenylhydrazone (FCCP, 2 μM), and antimycin A + rotenone (0.5 μM both). Basal oxygen consumption rate and mitochondrial Oxidative Phosphorylation System (OXPHOS) parameters were measured according to manufacturer's instructions of the Mito Stress test Kit (Seahorse Bioscience, Agilent Technologies, CA, USA). Results were normalized to protein quantified using a bicinchoninic acid-based protein assay (Thermo Fisher Scientific, Waltham, MA, USA) in each well.

For the Electron Flow Assay, Agilent instructions were followed. Briefly 5 μg of isolated mitochondria (BAT or IWAT) were resuspended in assay buffer (MAS-1; 70 mM sucrose, 220 mM mannitol, 2 mM HEPES, 1 mM EGTA, 10 mM KH_2_PO_4_, 5 mM MgCl_2_ and 0.2% BSA, pH 7.2), plus glutamate and malate (5 μM both). The mitochondria were seeded, centrifuged, and incubated 30 min at 37 °C. After basal readings, 2.2 μM rotenone was injected to inhibit electron transport complex I. In subsequent additions, succinate (5 mM) was added as a substrate of electron transport complex II followed by 40 μM antimycin A to inhibit complex III. Finally, 5 mM ascorbate and 10 mM N,N,N0,N0-Tetramethyl-p-phenylenediamine (TMPD) were added to measure the cytochrome *c* oxidase activity (complex IV).

For the coupling between the electrons, 5 μg of isolated IWAT mitochondria were resuspended in MAS-1 buffer, seeded, centrifuged, and incubated 30 min at 37 °C. To examine de degree of coupling between the electron transport chain and the oxidative phosphorylation machinery, coupled state was measured with the presence of substrate succinate (5 mM) and 2 μM rotenone (State II). State III was initiated with de addition of ADP (0.2 mM), meanwhile state IV was induced with the addition of oligomycin (1 μM). Maximal uncoupler-stimulated respiration was induced with the addition of 2 μM FCCP (State III). Finally, 0.5 μM antimycin A was added to block all mitochondrial respiration. The respiratory control ratio was calculated following the formula (RCR: State III/State IV).

For the norepinephrine (NE) OCR stimulation, differentiated BAT SVF brown adipocytes were incubated 1 h at 37 °C, in assay media with no BSA, before start of the readings. After basal measurements, NE (1 μM) was injected to measure adrenergically-induced uncoupled respiration. NE-induced uncoupled respiration curves were calculated by subtracting the basal OCR from the OCR values after NE treatment.

#### 2.3.14 Measurements of mitochondrial content and intracellular ROS

The mitochondrial DNA (mtDNA), an index of mitochondrial content (mtDNA copy number), was carried out in brown adipose tissue as previously validated and described [31]. Briefly, DNA was extracted using QIAzol® (Qiagen) protocol. Real-time PCR was performed to amplify the mitochondrial gene 16S which is exclusive of mtDNA, and a nuclear gene (mtDNA/nDNA ratio). In cultured brown adipocytes, Mitotracker Green (Thermo Fisher Scientific, Waltham, MA, USA) was used at 200 nM for 90 min, meanwhile Hoechst (Thermo Fisher Scientific, Waltham, MA, USA) was used at 2 μg/μl for 10 min followed by wash-out before imaging (Zeiss LSM 710 confocal microscope). Mitotracker Green was excited by a 488 nm laser, while 361 nm laser was used for detection of Hoechst. Imaging was performed with a 40× objective. The dye signal was quantified with the Image J analysis software and normalized to the number of cells (Hoechst dye). The ROS assay was performed as previously described [32]. Values were expressed as the ratio between the fluorescence of the oxidized CM-H2DCFDA (Thermofisher) and Hoechst, after subtraction of the signal of not-stained cells.

### 2.4 Statistical analysis

Statistical analysis was performed with GraphPad Prism 8.0 (GraphPad Software, CA, USA). Statistical parameters, including n values, are noted in figure legends. Data are presented as means ± standard error of the mean (SEM) and a p < 0.05 was considered significant. Statistical analysis of data with more than 2 groups was assessed with one-way ANOVA. Data with two groups was analysed by two-tailed Student's *t* test.

## 3 Results

### 3.1 White adipocyte SERCA2 is down-regulated in HFD-induced obese mice and in obese type-2 diabetic humans

**Figure 1:**
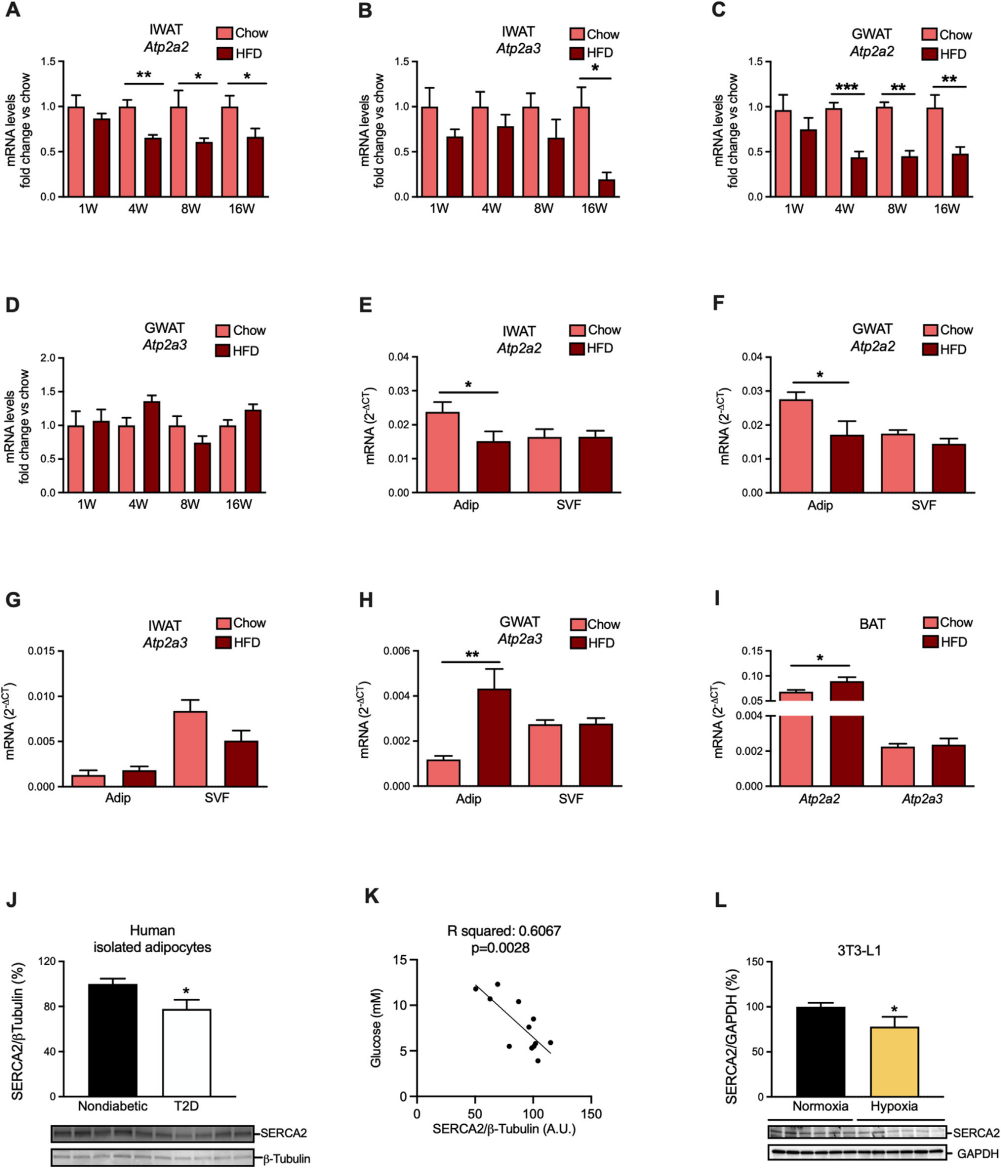
Adipocyte SERCA2 is down-regulated in high fat diet-induced obesity. *Serca2 (Atp2a2)* and *3 (Atp2a3)* mRNA levels in (**A–B**) IWAT and (**C–D**) GWAT at 1, 4, 8 and 16 weeks (W) high fat diet (HFD)- and chow-fed wild type mice (N = 6–10). (**E–H**) *Serca2 (Atp2a2) and 3 (Atp2a3)* mRNA levels in 10-week-HFD-fed mice IWAT and GWAT adipocytes or SVF (N = 6–10). (**I**) *Serca2 (Atp2a2)* and *3 (Atp2a3)* gene expression in 10-week-HFD-fed mice BAT (N = 4–6). (**J**) SERCA2 protein levels (expressed as % of nondiabetics) in human adipocytes isolated from patients diagnosed with type-2 diabetes (N = 6) [mean age 67 years (range 65–72); mean BMI 36.9 kg/m2 (range 30–43)] and from nondiabetic subjects (N = 6) [mean age 64 years (range 55–69); mean BMI 24.3 kg/m2 (range 23–27)]. (**K**) Correlation analysis for donor plasma glucose with SERCA2 in primary human adipocytes isolated from corresponding patients. (**L**) SERCA2 protein levels in 3T3-L1 adipocytes at normoxia vs. 24 h hypoxia, expressed as % of normoxia controls (N = 6/group). All values are expressed as mean ± SEM; ∗p < 0.05 and ∗∗p < 0.01.

By DNA microarray analysis, we found that *Serca2* (*Atp2a2*) and -3 (*Atp2a3*) are the most abundant *Serca*-paralogs in mouse inguinal, gonadal, and mesenteric WAT (IWAT, GWAT and MWAT) with expression both in the adipocyte and in the stromal vascular fraction (SVF), while *Serca1* (*Atp2a1*) was detected at extremely low levels. In isolated adipocytes, *Serca2* was the predominant paralog, although *Serca3* was also highly expressed (Suppl. Fig. 1A). SERCA2 expression and/or activity are reduced in liver, pancreatic islets, and cardiomyocytes in animal models of obesity and T2D [18,33,34]. This prompted us to investigate whether HFD-induced obesity reduces *Serca2* also in adipose tissue. Indeed, in both IWAT and GWAT *Serca2* mRNA levels were reduced from the fourth week in HFD-induced obese mice. In contrast, *Serca3* mRNA levels were unaltered in both fat depots until week 16 of HFD, when the expression was dramatically decreased in IWAT but not in GWAT (Figure 1A–D). This HFD-induced decrease in WAT *Serca2* was confirmed at the protein level (Suppl. Fig. 1B–C) and was due to reduced levels specifically in adipocytes, while SVF levels of *Serca2* remained unaffected (Figure 1E–F). The expression of *Serca*3 was unaltered in the isolated IWAT adipocyte fraction and tended to be downregulated in SVF from this adipose tissue depot (Figure 1G). *Serca*3 mRNA levels were however upregulated in GWAT adipocytes and unchanged in the SVF fraction (Figure 1H). In agreement with data from HFD-challenged adipocytes, 24 h palmitate treatment downregulated the SERCA2 protein levels in cultured 3T3-L1 adipocytes (Suppl. Fig. 1D). In contrast to findings in WAT, the brown adipose tissue (BAT) *Serca2* expression was slightly elevated in HFD-induced obese mice while *Serca*3 expression was unaltered (Figure 1I). To determine whether adipocyte SERCA2 is relevant also for human pathophysiology, we measured SERCA2 protein in subcutaneous adipocytes obtained from subjects with and without obesity-associated T2D. The SERCA2 levels were ∼20% lower in adipocytes from the obese individuals compared to the non-obese controls (Figure 1J) and correlated inversely with the plasma glucose levels of adipocyte donors (Figure 1K). As hypoxia downregulates SERCA2 in cardiomyocytes [[35], [36], [37]], we hypothesized that a similar mechanism may be at play in enlarged hypoxic adipocytes of insulin-resistant subjects [38,39]. Indeed, hypoxia (24 h) led to reduced SERCA2 protein and mRNA levels in 3T3-L1 adipocytes (Figure 1L, Suppl. Fig. 1E). *Serca3* was also reduced by hypoxia but was expressed at a 3000-fold lower level than *Serca2* (Suppl. Fig. 1F)*.* In conclusion, our results pinpoint *Serca2* as the major SERCA paralog that is downregulated in white adipocytes from both diet-induced obese mice and obese/T2D patients.

### 3.2 Inhibition of SERCA in adipocytes is associated with altered Ca^2+^ homeostasis and defective adiponectin exocytosis

**Figure 2:**
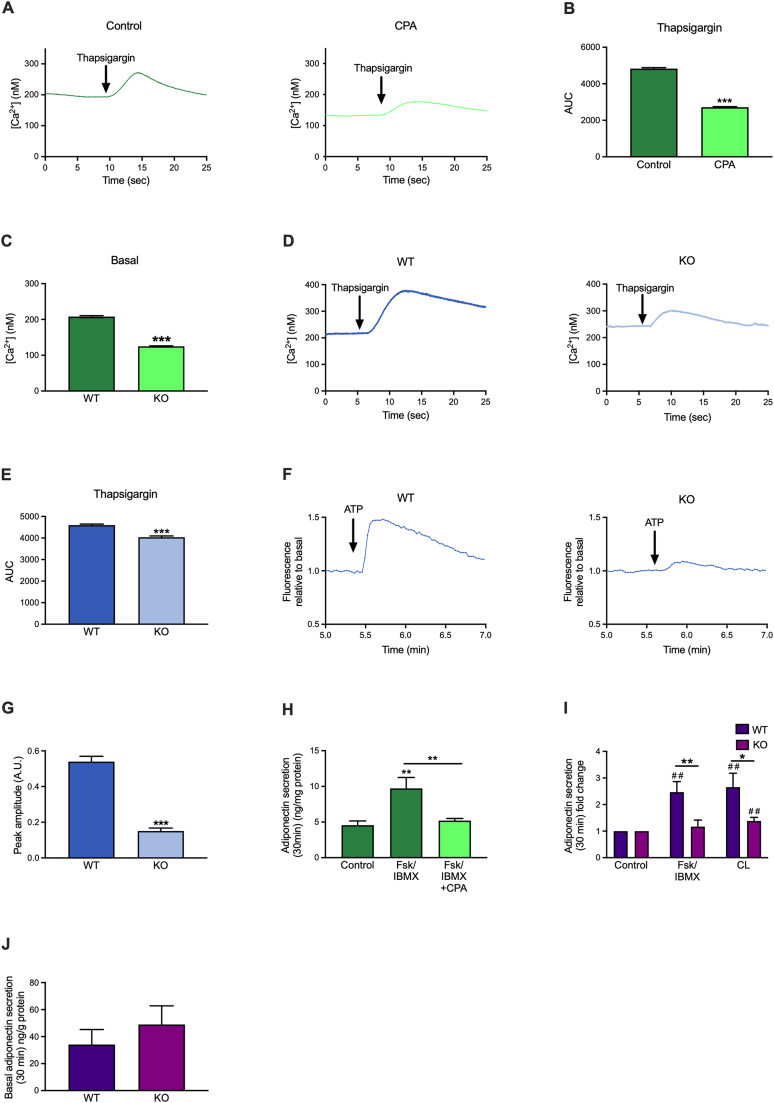
Inhibition of SERCA in adipocytes is associated with altered Ca^2+^ homeostasis and defective adiponectin exocytosis. (**A**) Example traces of typical [Ca^2+^]_i_ responses in 3T3-L1 using Fura-2 and exposed to slow flow of thapsigargin (1 μM) previously treated with cyclopiazonic acid (CPA) or vehicle for 4 h. (**B**) Area under the curve (AUC) of the [Ca^2+^] response in 3T3-L1 using Fura-2 and exposed to slow flow of thapsigargin (1 μM) previously treated with CPA or vehicle for 4 h. (**C**) Basal [Ca^2+^] in 3T3-L1 using Fura-2 treated with CPA or vehicle for 4 h. (**D**) Example traces of typical [Ca^2+^]_i_ responses in differentiated wildtype (WT) or SERCA2 knockout (KO) IWAT SVF cells using Fura-2 and exposed to slow flow of thapsigargin (1 μM). (**E**) Area under the curve (AUC) of the [Ca^2+^] response in differentiated WT or adipocyte-specific SERCA2 KO IWAT SVF cells using Fura-2 and exposed to slow flow of thapsigargin (1 μM). (**F**) Example traces in response to ATP of cytoplasmic Ca^2+^ in differentiated WT or SERCA2 KO IWAT SVF cells using Fluo-4 dye. (**G**) ATP peak amplitude of the cytoplasmic Ca^2+^ in differentiated IWAT SVF cells using Fluo-4 dye. (**H**) Total adiponectin secretion during 30 min treatment with Forskolin (10 μM; Fsk/IBMX) together with IBMX (200 μM) in 3T3-L1 with CPA or vehicle for 24 h. (**I**) Stimulated and (**J**) basal adiponectin secretion (30 min) in primary IWAT adipocytes isolated from adipocyte-specific SERCA2 KO mice. Cells were incubated with (10 μM; Fsk/IBMX) together with IBMX (200 μM) or beta-3 adrenergic receptor agonist CL316,243 (10 μM, CL, ##p < 0.01 for the difference between Fsk/IBMX or CL vs. control). Values (N = 6–10) are expressed as mean ± SEM; ∗p < 0.05, ∗∗p < 0.01, and ∗∗∗p < 0.001.

We next tested how SERCA inhibition affects the adipocyte Ca^2+^ homeostasis by performing ratiometric time-lapse recordings of intracellular Ca^2+^ concentrations. Pre-treatment of 3T3-L1 adipocytes with 20 μM of the SERCA inhibitor cyclopiazonic acid (CPA) reduced the ER calcium store by ∼50%, as shown by the diminished Ca^2+^ increase in response to acute addition of thapsigargin (another SERCA inhibitor) during the recording (Figure 2A–B). A similar reduction of stored ER calcium was found in cultured adipocytes genetically ablated for SERCA2 (Figure 2C–E). The reduced Ca^2+^ stores in SERCA2-ablated adipocytes were associated with the transcriptional upregulation in IWAT of two key players of cellular Ca^2+^ influx mediated via store-operated Ca^2+^ entry (SOCE): the stromal interaction molecule 1 (*Stim1*) that recognized when ER Ca^2+^ levels are low, and the calcium release-activated calcium channel protein 1, *Orai1* [40] (Suppl. Fig. 2A). We also observed a downregulation of the Ryanodine Receptor channel (*Ryr3*) that mediate Ca^2+^ release from ER to cytosol [41] (Suppl. Fig. 2A).

It has been demonstrated that the extracellular application of ATP rapidly elevates cytosolic Ca^2+^ in adipocytes via purinergic P2Y2 receptors and STIM1/ORAI1-mediated activation of SOCE [42,43]. We thus tested the effect of ATP on cultured SERCA2-ablated adipocytes. Mitochondria can accumulate a 10-fold higher concentration of Ca^2+^ than that measured in the cytosol and Ca^2+^ is transferred between ER and the mitochondria via mitochondria-associated membranes (MAMs), specialised regions of communication between the two organelles [44]. Mitochondrial Ca^2+^ levels were therefore measured in parallel. In agreement with [42,43], extracellular addition of ATP (100 μM) elevated [Ca^2+^]_i_ in control adipocytes while the ATP-induced [Ca^2+^]_i_ peak was reduced in SERCA2-deficient adipocytes (Figure 2F–G). The ATP-triggered mitochondrial Ca^2+^ elevations were similarly affected in these knockout adipocytes (Suppl. Fig. 2B–C). This finding was associated with a transcriptional upregulation of the mitochondrial calcium uniporter (*Mcu*) that mediates electrophoretic Ca^2^⁺ uptake into the mitochondrial matrix [45] in IWAT of adipocyte-specific SERCA2 deficient mice (Suppl. Fig. 2A). Thus, SERCA2 regulates Ca^2+^ in both the mitochondria and the ER of white adipocytes.

To further explore the importance of SERCA for metabolic regulation, we studied the role of these Ca^2+^ pumps in adiponectin secretion. Ca^2+^ is important for sustained adiponectin secretion [12,46] and both mitochondrial dysfunction and ER stress have been linked to reduced adiponectin production in adipocytes [47,48]. The effect of SERCA inhibition on regulated adiponectin exocytosis has however not previously been studied. CPA treatment induced ER stress in 3T3-L1 adipocytes (as verified by increased *Bip* and *Xbp1s* expression), reaching significance after 4 h (Suppl. Fig. 2D–E). Adiponectin secretion stimulated by a combination of forskolin and IBMX (elevates intracellular cAMP and also slightly increases Ca^2+^ in adipocytes; [13]) was blunted in CPA-treated cells (Figure 2H) whereas basal (unstimulated) adiponectin release was unaffected (not shown). Adiponectin secretion triggered by forskolin/IBMX or the beta 3 adrenergic receptor agonist CL-316,243 (CL) was similarly blunted also in primary adipocytes genetically ablated for SERCA2 (Figure 2I). Basal adiponectin release was not altered in SERCA2 null adipocytes compared to wild type (Figure 2J).

### 3.3 Adipocyte-specific SERCA2 knockout causes ER stress in IWAT and BAT

**Figure 3:**
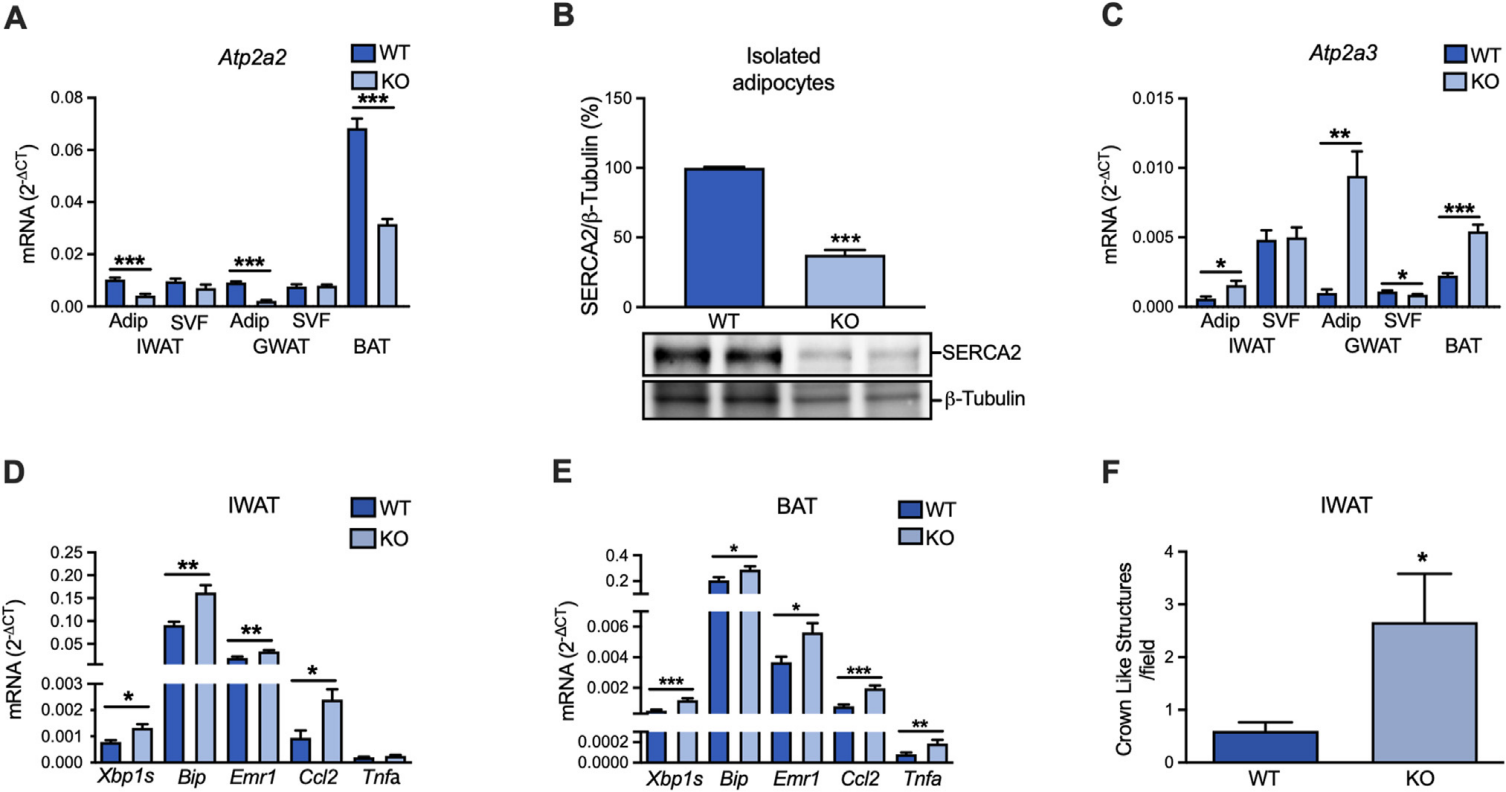
Adipocyte-specific SERCA2 ablation leads to elevated markers of ER stress and inflammation in IWAT and BAT. (**A**) *Serca2 (Atp2a2)* gene expression in adipocytes and SVF of IWAT, GWAT and BAT in chow diet-fed mice. (**B**) SERCA2 protein levels (expressed as % of WT) in isolated WT and adipocyte-specific SERCA2 KO IWAT adipocytes. (**C**) *Serca3 (Atp2a3)* gene expression in adipocytes and SVF of IWAT, GWAT and BAT in chow diet-fed mice. (**D–E**) ER stress and inflammation gene expression markers in IWAT and BAT chow diet-fed mice. (**F**) Crown like structures in IWAT of chow or HFD-fed mice. Values (N = 6–10) are expressed as mean ± SEM; ∗p < 0.05, ∗∗p < 0.01, and ∗∗∗p < 0.001.

To define the role of SERCA2 for adipocyte functionality and whole-body metabolic health, we generated an adipocyte-specific *Serca2* knockout mouse model. The knockouts displayed ∼60–70% reduced *Serca2* levels in IWAT, GWAT and BAT, while the SVF *Serca2* expression was unaffected (Figure 3A). SERCA2 protein levels were reduced to a similar degree in isolated *Serca2* knockout adipocytes (Figure 3B). There was a compensatory upregulation of *Serca3* expression in the adipocyte fraction of WAT and BAT, and this *Serca3* increase was particularly pronounced in GWAT adipocytes (Figure 3C). Downregulation of *Serca2* was associated with increased expression of the UPR-markers *Xbp1s* and *Bip* and the inflammatory markers *Emr1* (*F4/80*), *Ccl2* (*Mcp1*) and *Tnfα* in IWAT and BAT, indicating ER stress (Figure 3D–E). In support of a higher degree of IWAT inflammation, there was also an increased abundance of crown-like structures (Figure 3F). Interestingly, the GWAT expression of UPR and inflammation markers was unaffected by knockdown of *Serca2* (Suppl. Fig. 3), possibly due to the compensatory elevation of *Serca3* in this fat depot (Figure 3C).

### 3.4 Adipocyte-specific SERCA2-deficient mice are mildly lipodystrophic and display whole-body metabolic dysfunction

**Figure 4:**
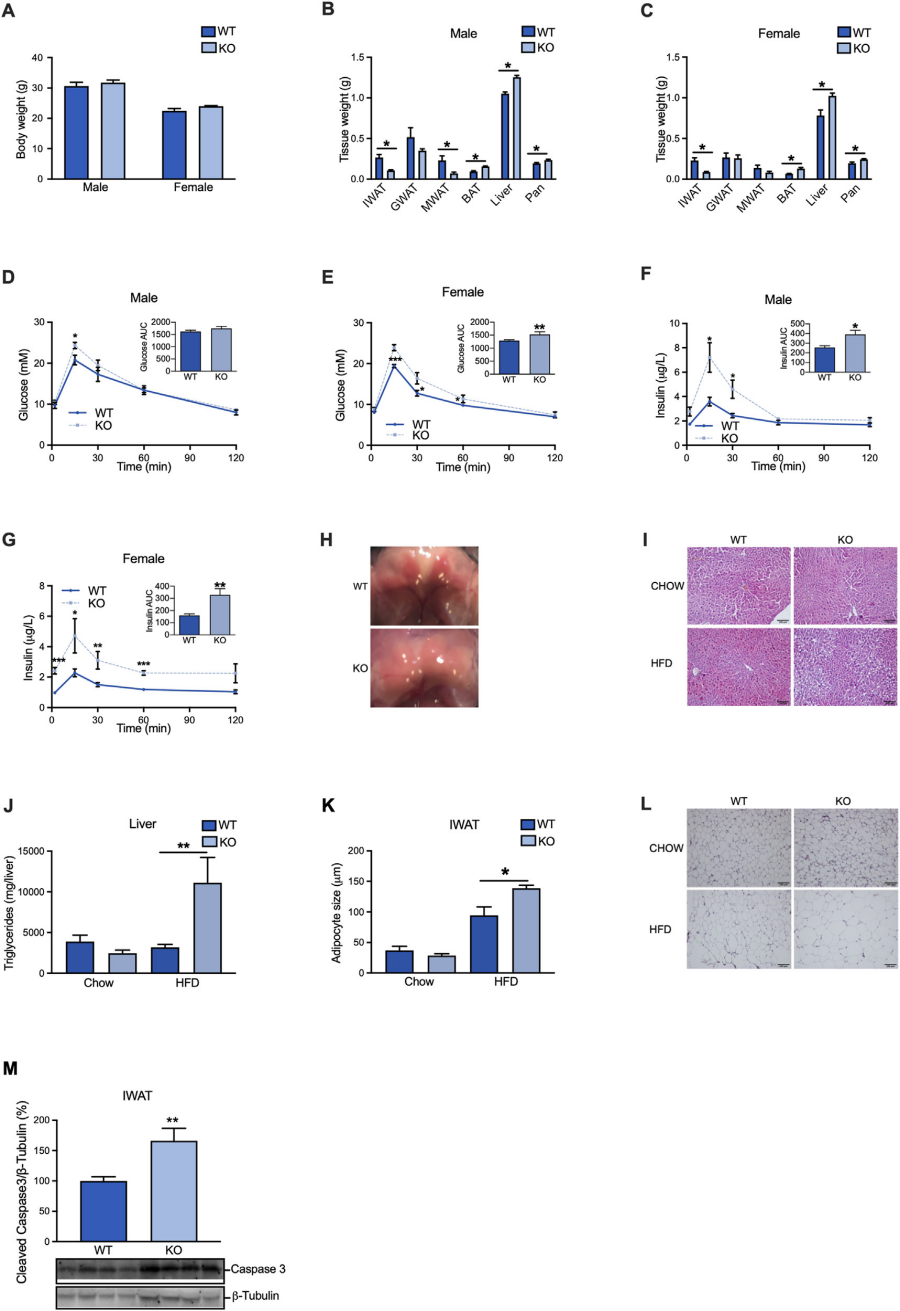
Adipocyte-specific SERCA2 deficient mice are lipodystrophic and display impaired whole-body metabolic function. (**A**) Body weight, (**B–C**) IWAT, GWAT, MWAT, BAT, liver, and pancreas weight of WT and adipocyte-specific SERCA2 KO chow-fed male and female mice at 8 weeks of age (N = 6–10). (**D–G**) Circulating glucose and insulin levels at indicated time points and AUC in response to oral glucose load in chow-fed male and female WT and adipocyte-specific SERCA2 KO mice. (**H**) Representative images of BAT from WT and adipocyte-specific SERCA2 KO mice. (**I**–**J**) Liver triglyceride content and (**K–L**) adipocyte size in IWAT from mice on chow or 10-week-HFD. (**M**) IWAT cleaved caspase3 protein levels (expressed as % of WT) in chow-fed WT and adipocyte-specific SERCA2 KO mice. Values (N = 6–10) are expressed as mean ± SEM; ∗p < 0.05 and ∗∗p < 0.01.

Male and female adipocyte-specific SERCA2 knockout mice grew normally after weaning, but displayed reduced WAT mass, while their liver, pancreas and BAT masses were increased (Figure 4A–C). Heart (0.183 ± 0.006 vs. 0.186 ± 0.007 g in males) and skeletal muscle (Gastrocnemius; 0.202 ± 0.008 vs. 0.212 ± 0.015 g in males) masses were unchanged. This lipodystrophic phenotype was associated with impaired whole-body metabolic function, as judged by slightly reduced glucose tolerance and hyperinsulinemia (Figure 4D–G), as well as a trend towards increased glucagon levels (2.61 ± 0.33 vs. 4.44 ± 0.97 pmol/L, p = 0.122). The enlarged pancreata of adipocyte-specific SERCA2 knockout mice displayed normal morphology (judged by haematoxylin and eosin staining), and the islet density and size distribution per analysed section were similar between genotypes (Suppl. Fig. 4A–C). Insulin secretion induced by high glucose, arginine and tolbutamide was elevated in adipocyte-specific SERCA2 knockouts (Suppl. Fig. 4D). The increased BAT weight appeared to primarily result from increased fat deposition, as judged by the whiteish BAT appearance (Figure 4H). The hepatic triglyceride content was similar between genotypes (Figure 4I–J).

When fed HFD (to induce obesity), control and adipocyte-specific SERCA2 knockout mice exhibited similar weight gain (Suppl. Fig. 4E), and the difference in WAT, BAT and liver mass persisted between genotypes (Suppl. Fig. 4F). In this metabolically challenged setting, the hepatic lipid deposition was increased in the knockouts (Figure 4I–J). The glucose intolerance was aggravated by HFD, but the differences between genotypes were similar to those observed at chow conditions (Suppl. Fig. 4G–J).

To elucidate the underlying reasons for the reduced WAT mass, we measured IWAT adipocyte size. During chow conditions, the adipocyte-specific SERCA2 knockouts displayed smaller IWAT adipocytes (although the difference did not reach significance). Despite ∼60% decreased IWAT weight, the SERCA2 knockout adipocytes of HFD-fed mice were ∼50% larger than littermate wild type control adipocytes (Figure 4K–L). This increase in average adipocyte size was primarily due to a doubling of the percentage of larger adipocytes (>175 μm in diameter), while the fraction of smaller adipocytes was lower (Suppl. Fig. 4K). These data indicate that SERCA2 deficiency does not lead to impaired lipid storage capacity of an individual adipocyte, but that there is an increase in HFD-induced adipocyte death (as implied by the increased occurrence of crown-like structures, Figure 3F) and/or a decreased formation of new adipocytes in these mice. Given that only adiponectin-expressing cells (i.e. mature adipocytes), are SERCA2-deficient in this mouse model and that HFD-induced expansion largely depends on hypertrophy [49], we argued that increased adipocyte death is more likely to explain the fewer but larger IWAT adipocytes in HFD-fed mice. Indeed, the IWAT levels of cleaved caspase 3 were increased in adipocyte-specific SERCA2 knockout mice (Figure 4M) and the adipogenic capacity was similar between genotypes as judged by *ex vivo* differentiation of IWAT SVF cells (Suppl. Fig. 4L).

### 3.5 Levels and secretion of adipocyte hormones are altered in adipocyte-specific SERCA2 knockout mice

**Figure 5:**
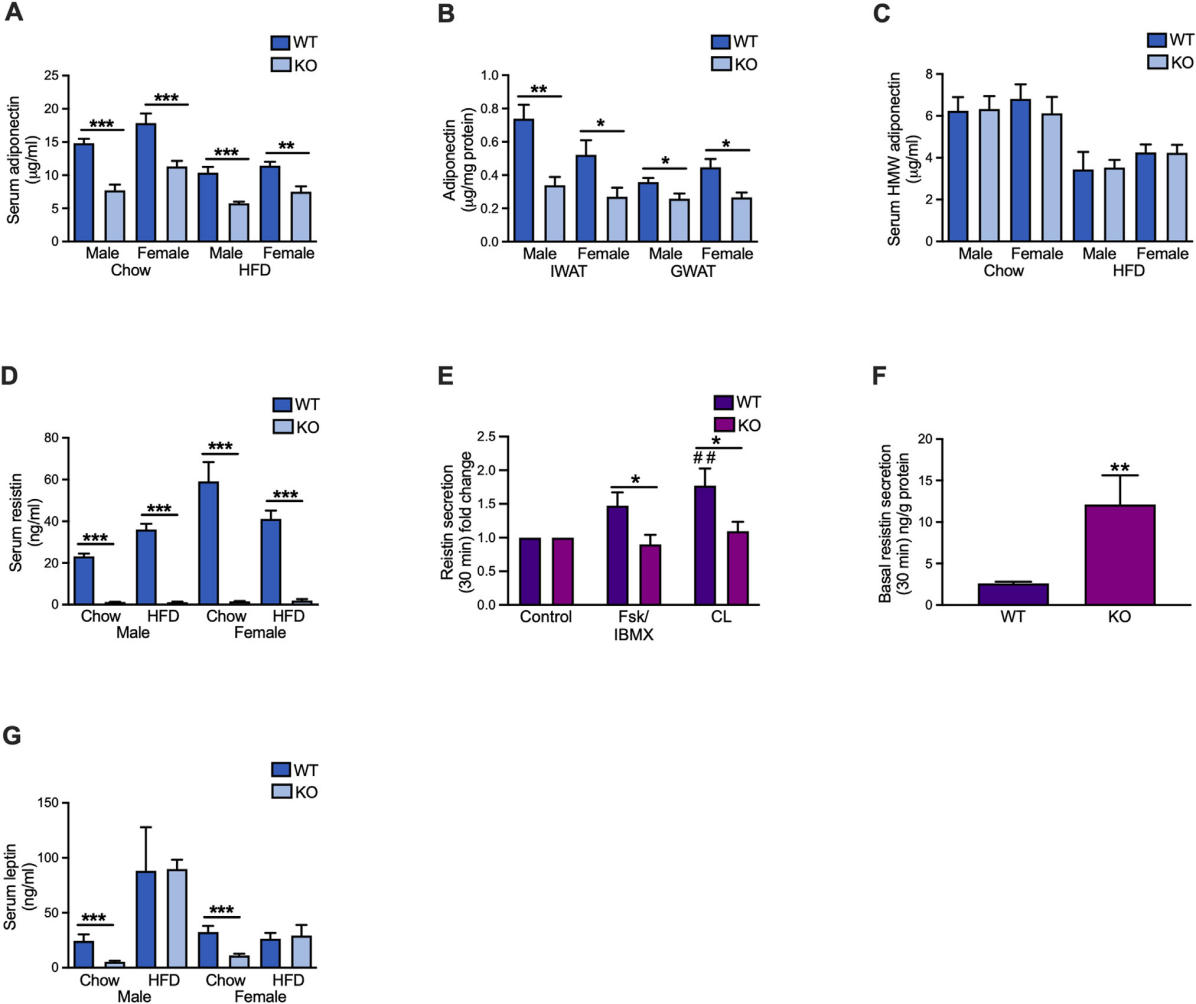
Adipocyte hormones are altered in adipocyte-specific SERCA2 knockout mice. (**A**) Serum total adiponectin levels, (**B**) IWAT and GWAT total adiponectin levels, (**C**) serum HMW-adiponectin levels, (**D**) serum resistin levels in male and female chow and 10-week-HFD-fed WT and adipocyte-specific SERCA2 KO mice. (**E**) Stimulated and (**F**) basal resistin secretion (30 min) in primary IWAT adipocytes isolated from adipocyte-specific SERCA2 KO knockout. Cells were incubated with (10 μM; Fsk/IBMX) together with IBMX (200 μM) or beta-3 adrenergic receptor agonist CL316,243 (10 μM, CL). (**G**) Serum leptin in male and female chow and 10-week-HFD-fed WT and adipocyte-specific SERCA2 KO mice. All values (N = 6–10) are expressed as mean ± SEM; ∗p < 0.05, ∗∗p < 0.01, and ∗∗∗p < 0.001.

As SERCA2-deficient adipocytes display abrogated regulated release of adiponectin (Figure 2H–I), we determined whether adipocyte-specific SERCA2 knockouts have altered levels of the adipose tissue hormones adiponectin, resistin and leptin. Both male and female adipocyte-specific SERCA2 knockout mice had much reduced levels of circulating adiponectin and this was associated with decreased adiponectin protein and gene expression in IWAT and GWAT (Figure 5A–B, Suppl. Fig. 5A). In contrast, serum HMW adiponectin levels were similar between adipocyte-specific SERCA2 knockouts and littermate wild type controls. However, HFD-feeding reduced both total and HMW adiponectin in both genotypes (Figure 5C). Because of the unaltered HMW adiponectin levels, the HMW to total adiponectin ratio was significantly higher in the male knockouts with a similar trend in females (Suppl. Fig. 5B). The ER chaperones disulphide-bond A oxidoreductase-like protein (*DsbA-L*), ER membrane-associated oxidoreductase (*Ero1-Lα*) and its associated protein *Erp44* have been identified as critical players in the assembly and secretion of HMW adiponectin [50,51]. We found increased gene expression of *Ero1lα* in IWAT from both male and female knockouts, and higher gene expression of *Erp44* in IWAT from the female SERCA2 null mice (Suppl. Fig. 5C).

Resistin has been suggested to contribute to the pathogenesis of metabolic diseases, although its exact role is not fully elucidated [[52], [53], [54]]. Similar to adiponectin, resistin has a complex structure with multimeric assembly [55] and the two hormones are in mouse adipocytes co-released from the same vesicles [56]. As expected, circulating resistin levels were dramatically reduced both at chow and HFD-fed conditions (Figure 5D) and the stimulated resistin secretion was blunted in primary SERCA2-ablated adipocytes, while the basal release was increased (Figure 5E–F); IWAT and GWAT resistin (*Retn*) mRNA levels were also much reduced (Suppl. Fig. 5D).

In contrast to adiponectin and resistin, leptin is released as a monomer in proportion to the degree of adiposity. We therefore expected a less potent effect of SERCA2 ablation on leptin levels. In line with this assumption, circulating leptin levels as well as IWAT and GWAT leptin (*Lep*) mRNA levels were similar between genotypes in HFD-fed mice (Figure 5G and Suppl. Fig. 5E). Circulating leptin and IWAT *Lep* mRNA levels were however reduced in chow-fed knockouts, in agreement with their lower fat mass (Figure 5G and Suppl. Fig. 5E).

### 3.6 Opposite effect of adipocyte-specific SERCA2 ablation on white and brown adipose tissue glucose uptake

**Figure 6:**
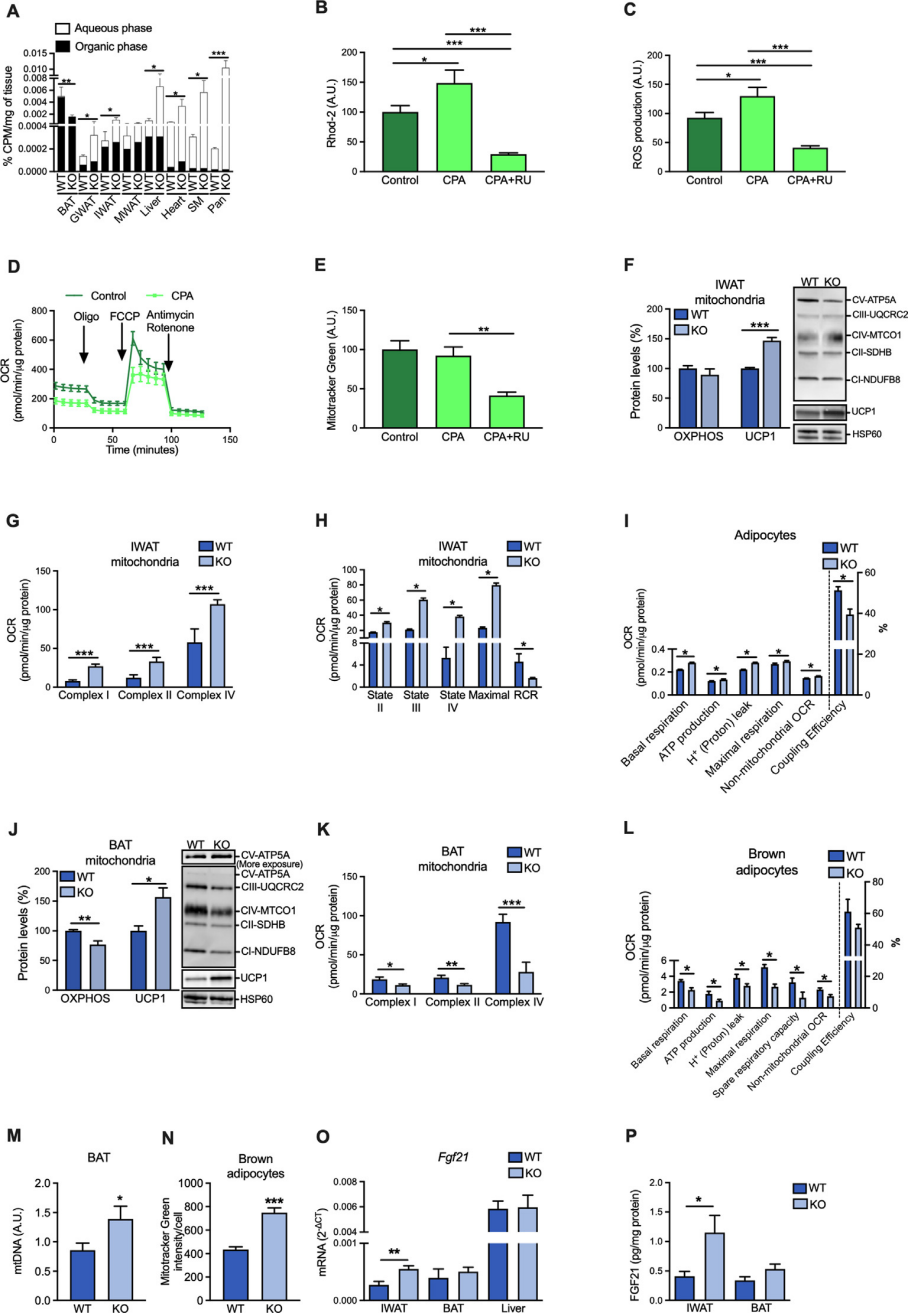
Opposite effect of adipocyte-specific SERCA2 ablation on white and brown adipose tissue glucose uptake and mitochondrial function. (**A**) Total glucose uptake in chow-fed WT and adipocyte-specific SERCA2 KO mice. (**B**) Mitochondrial calcium measured with Rhod-2 dye and (**C**) ROS production measured with CM-H_2_DCF-DA dye in 3T3-L1 treated with vehicle or CPA with or without the mitochondrial calcium uptake inhibitor RU for 1 h. (**D**) Oxygen consumption rate (OCR) in 3T3-L1 treated with CPA or vehicle for 4 h. (**E**) Mitochondrial mass measured with Mitotracker Green dye. (**F**) Total OXPHOS (Complex I–V) and UCP1 protein levels (expressed as % of WT) in isolated mitochondria IWAT in chow-fed WT and adipocyte-specific SERCA2 KO mice. (**G**) OCR of the different mitochondrial complexes and (**H**) respiratory states analysed in isolated IWAT mitochondria from chow-fed WT and adipocyte-specific SERCA2 KO mice. (**I**) OCR parameters calculated in adipocytes differentiated from IWAT SVF from chow-fed WT and adipocyte-specific SERCA2 KO mice. (**J**) Total OXPHOS (Complex I–V) and UCP1 protein levels (expressed as % of WT) in isolated BAT mitochondria from chow-fed WT and adipocyte-specific SERCA2 KO mice. (**K**) OCR of the different mitochondrial complexes in isolated BAT mitochondria from chow-fed WT and adipocyte-specific SERCA2 KO mice. (**L**) OCR parameters in brown adipocytes differentiated from BAT SVF from chow-fed WT and adipocyte-specific SERCA2 KO mice. (**M**) BAT mitochondrial DNA (mtDNA) levels in chow-fed WT and adipocyte-specific SERCA2 KO mice, and (**N**) Mitotracker Green dye intensity in brown adipocytes differentiated from BAT SVF from chow-fed WT and adipocyte-specific SERCA2 KO mice. (**O**) *Fgf21* expression in IWAT, BAT and liver in chow-fed WT and adipocyte-specific SERCA2 KO mice. (**P**) FGF21 protein levels in IWAT and BAT from chow-fed WT and adipocyte-specific SERCA2 KO mice. All values (N = 3–6) are expressed as mean ± SEM; ∗p < 0.05, ∗∗p < 0.01, and ∗∗∗p < 0.001.

We speculated that the phenotype of SERCA2 knockout mice is due to the systemic impact of impaired metabolic function of white and brown adipocytes. To investigate this, we used [^14^C]-labelled glucose to measure tissue glucose uptake and *de novo* lipogenesis 2 h after an oral glucose load. Contrary to our expectations, we found that the glucose uptake was increased in WAT (represented by IWAT, GWAT and MWAT), while only BAT displayed a clear reduction in glucose uptake (Figure 6A). The enhanced glucose uptake in knockout WAT was primarily due to increased [^14^C]-counts in the aqueous phase, likely reflecting glucose oxidation (Figure 6A). In contrast, [^14^C]-counts in the organic phase (representing *de novo* lipogenesis) dominated in BAT (Figure 6A). Glucose uptake was also augmented in liver, heart, skeletal muscle, and pancreas, largely due to an increase in the aqueous phase (Figure 6A). Thus, the impaired glucose uptake in BAT appears to be largely compensated for by increased glucose-stimulated insulin release from pancreas leading to enhanced glucose uptake in insulin-sensitive tissues. This provides an explanation for the nearly normal whole-body glucose tolerance (Figure 4D).

### 3.7 Pharmacological inhibition of SERCA is associated with mitochondrial dysfunction in 3T3-L1 adipocytes

The increased glucose uptake and metabolism in WAT from adipocyte-specific SERCA2 null mice urged us to investigate the effect of SERCA inhibition on mitochondrial function. Imaging of Rhod-2 loaded 3T3-L1 adipocytes showed that the basal mitochondrial Ca^2+^ accumulation was increased by CPA treatment (Figure 6B), indicating transfer of Ca^2+^ from the ER to the mitochondria [44]. The increase in mitochondrial Ca^2+^ was accompanied by a higher ROS production and reduced oxygen consumption rate (OCR; Figure 6C–D). As expected, administration of the mitochondrial Ca^2+^ uptake inhibitor RU360 reduced both basal and CPA-induced mitochondrial Ca^2+^ and ROS levels (Figure 6B–C). As shown in Figure 6E, the cell mitochondrial content (judged by Mitotracker green staining) was unchanged by CPA but reduced by RU360 treatment. Taken together, our results indicate that acutely disturbed intracellular Ca^2+^ homeostasis affects the mitochondrial function in adipocytes.

### 3.8 Adipocyte-specific SERCA2-deficient mice display altered mitochondrial dysfunction in IWAT and in BAT

Lack of SERCA2 in white adipocytes may result in enhanced metabolism (as indicated by the increased WAT glucose uptake) through a mitohormesis-like process. We explored this possibility by analysing the expression of browning markers and found a trend for increased IWAT *Ucp1* levels in the adipocyte-specific SERCA2 knockout mice, while the browning markers *Prdm16* and *Dio2* were downregulated (Suppl. Fig. 6A). Notably, UCP1 protein levels in isolated IWAT mitochondria were ∼1.5-fold higher in SERCA2 ablated mice than in littermate wild type controls, while electron transport chain protein levels were similar between genotypes (Figure 6F). Furthermore, the OCR of isolated IWAT mitochondria was much higher (Figure 6G–H). This increase in mitochondrial OCR was primarily due to a lower coupling efficiency in SERCA2-deficient adipocytes (Figure 6H). To test if this effect of SERCA2 ablation on mitochondrial function is cell autonomous, we analysed the mitochondrial function in *in vitro* differentiated adipocytes from IWAT SVF. Indeed, also cultured SERCA2-deficient adipocytes displayed elevated uncoupled OCR and the ATP production-linked OCR was slightly increased (Figure 6I).

The altered expression of browning markers was similar in BAT and IWAT; BAT *Prdm16* and *Dio2* levels were reduced, while *Ucp1* was unchanged in adipocyte-specific SERCA2 knockouts compared to littermate wild type controls (Suppl. Fig. 6B). Moreover, the level of total electron transport proteins was reduced along with increased UCP1 protein levels in isolated mitochondria from BAT of adipocyte-specific SERCA2 knockout mice (Figure 6J). In sharp contrast to SERCA2-deficient white adipocytes, mitochondria of BAT adipocyte-specific SERCA2 knockout mice displayed lower OCR (Figure 6K). Accordingly, OCR was decreased in cultured SERCA2-deficient brown adipocytes, as shown by the reduction of basal and ATP-production-linked respiration and other key mitochondrial respiration parameters, (Figure 6L). Surprisingly, this reduction in mitochondrial OCR was associated with elevated markers of mitochondrial content in both BAT and differentiated brown adipocytes (Figure 6M–N). However, the capacity to increase the respiration in response to norepinephrine was unaffected (Suppl. Fig. 6C).

Several studies have shown that ER stress can lead to an adaptive increase in fibroblast growth factor 21 (FGF21) [57,58], a metabolic hormone capable of inducing thermogenesis in BAT and promoting a brown fat-like thermogenic program in white adipocytes [59,60]. In line with the increased UPR marker expression (Figure 3D), we found that the expression of FGF21 was elevated in adipocyte-specific SERCA2 knockout IWAT (Figure 6O–P). However, such an ER-stress/UPR-associated induction of FGF21 was not observed in BAT (Figure 3, Figure 6O–P). As expected, the hepatic *Fgf21* mRNA and serum protein levels were similar between genotypes (Figure 6O, Suppl. Fig. 6D). Thus, our data suggest that genetic ablation of SERCA2 leads to different adaptations in white and brown adipocytes.

## 4 Discussion

Here we show that diet-induced obesity in mice, T2D in humans, and exposure to palmitate or hypoxia are associated with lower SERCA2 levels in white adipocytes. SERCA2-deficient white adipocytes display altered calcium homeostasis, hormone release and mitochondrial function, and adipocyte-specific SERCA2 knockout mice present with hyperinsulinemia, glucose intolerance and lower adiponectin levels, likely driven by both white and brown adipocyte dysfunction. In brief, the WAT of this mouse model fails to expand properly, presumably because of increased adipocyte death. However, the remaining SERCA2-deficient white adipocytes show accelerated metabolism, as judged by increased glucose uptake and increased OCR. In contrast, BAT is enlarged but less metabolically active, as demonstrated by its whiter appearance, decreased glucose uptake, and decreased OCR. Furthermore, this mouse model illustrates how genetically normal tissues to some extent can compensate for adipose tissue defects. For instance, the glucose uptake is dramatically increased in liver, heart, skeletal muscle and pancreas, and the glucose-stimulated insulin release is enhanced, both *in vivo* and *ex vivo* in isolated islets.

### 4.1 Maintained Ca^2+^ dynamics and ER function are essential for adipocyte hormone secretion

The ER is the first compartment of the secretory pathway, where proteins are folded, oligomerized and finally packaged into vesicles that continue their route towards the plasma membrane. Protein folding and oligomerization in ER is controlled by Ca^2+^-dependent chaperones [3] and obesity-associated ER stress has been shown to impair adipocyte hormone secretion [1]. In agreement with this, we found that circulating levels of adiponectin are reduced in the adipocyte-specific SERCA2 null mice (Figure 5A). However, the HMW form of adiponectin remained unaffected in serum from SERCA2 ablated mice (Figure 5C). A few studies suggest that reduced circulating levels of specifically the HMW form of adiponectin is connected to development of obesity-associated metabolic disease and that higher-order adiponectin complexes protect from progress to T2D [61,62]. Thus, the adiponectin aberrations observed in the adipocyte-specific SERCA2 null mice do not fully concur with adiponectin alterations reported in metabolic disease. The found increase in *Erp44* and *Ero1-Lα*, two ER chaperones belonging to the protein-disulphide isomerase family may underly this difference [63]. Disulphide bonds form and stabilize the higher-order adiponectin complexes [64] and *Erp44* and *Ero1-Lα* are critically important for the proper ER-located modifications and the secretion of adiponectin. Cycloheximide-induced inhibition of protein synthesis, which leads to retention of proteins within the ER, resulted in production of more HMW adiponectin, at the expense of smaller adiponectin forms [65]. Thus, we suggest that disturbed Ca^2+^ dynamics and ER stress in SERCA2-ablated adipocytes result in retention of adiponectin within the ER. This, together with the concomitant upregulated expression of *Erp44* and *Ero1-Lα*, promote the assembly of HMW adiponectin at the expense of smaller adiponectin forms. Furthermore, circulating adiponectin levels may not only reflect the secretory function of individual adipocytes, but rather the sum of the functionality of different fat depots (as well as effects of negative feedback and hepatic clearance). GWAT and IWAT display different adipocyte hormone release patterns [66,67] and in obese subjects, small sized visceral adipocytes were positively correlated to serum HMW adiponectin [68]. The finding that SERCA2 knockout GWAT adipocytes displayed a dramatic increase in *Serca3* expression (Figure 3B) points to a less pronounced phenotype in this fat depot. Thus, the higher HMW/total adiponectin in adipocyte-specific SERCA2 knockout mice, may result also from GWAT adipocytes remaining more functional and contributing more to the levels of higher-order adiponectin complexes.

The circulating levels of resistin are dramatically reduced in the adipocyte-specific SERCA2 knockouts (Figure 5D). This is not surprising as resistin, like adiponectin, circulates in differently sized molecular forms [69] and maintained ER chaperone activity is likely essential for its assembly and secretion. Moreover, it has been suggested that resistin itself functions as a chaperone and is retained within the ER during ER stress to protect from cell apoptosis [70]. Leptin, on the other hand, is released from adipocytes as molecular monomers and yet circulating leptin levels are also lower in adipocyte-specific SERCA2 knockouts. We attribute this difference in leptin levels primarily to the reduced adipose tissue mass in the adipocyte-specific SERCA2 knockouts [71]. In support of this, serum leptin levels were similar in HFD-fed wild type and adipocyte-specific SERCA2 knockout mice, the latter with increased adipocyte size which is strongly associated with increased leptin secretion and levels [72,73]. Thus, our study indicates that loss of adipocyte SERCA2 in obesity/T2D can mediate alterations in adipokine levels, although genetic SERCA2 ablation causes more dramatic effects than those observed in metabolic disease where the reduction in adipocyte SERCA2 is less dramatic (Figure 1E, F, J).

### 4.2 Genetic ablation of SERCA2 impairs calcium homeostasis and induces different thermogenic adaptations in white and brown adipocytes

Adipocyte-specific SERCA2 knockout BAT and IWAT both display reduced expression of *Prdm16* and *Dio2* (Suppl. Fig. 6A–B) and increased mitochondrial UCP1 levels (Figure 6F, J). However, BAT glucose uptake and mitochondrial respiration are reduced while we observed accelerated metabolism in IWAT of the adipocyte-specific SERCA2 knockout mice (Figure 6A, G, H, K). These differences in mitochondrial respiration between genotype are seen also in cultured white and brown adipocytes (Figure 6I, L) arguing for a cell autonomous mechanism. Similar differences in IWAT and BAT mitochondrial responses to stressors have been reported previously [26,74,75]. Moreover, the OCR was recently reported to increase in subcutaneous adipocytes from insulin resistant compared to insulin sensitive obese subjects [76], which is consistent with our findings (Figure 1, Figure 6G–H). In contrast to genetic SERCA2 ablation, acute pharmacological SERCA inhibition in 3T3-L1 adipocytes increases the mitochondrial Ca^2+^ levels associated with increased ROS production and reduced OCR (Figure 6B–D). Thus, the browning-like effect in SERCA2-deficient adipocytes appears to be part of an adaptive ER stress-induced mitohormetic that does not occur upon acute SERCA inhibition. Based on several reports [[77], [78], [79], [80], [81]], we propose that the upregulation of UCP1 (Figure 6F, J) is triggered by increased mitochondrial ROS levels, to suppress further increases in the ROS production and Ca^2+^ overload in SERCA2-deficient adipocytes. It is also possible that the already high levels of UCP1 and endogenous antioxidant enzymes prevent the mitohormetic response in BAT, as has been suggested previously [75]. The different adaptations of white and brown adipocytes to SERCA2 ablation may also be explained by differential induction of FGF21; FGF21 was increased in IWAT but not in BAT (Figure 6N). However, the reduction in mitochondrial metabolism may also signpost a specific role of SERCA2 in mitochondrial function of brown adipocytes that cannot be compensated for by SERCA1 and 3. Indeed, the SERCA-ryanodine receptor (RyR)-pathway has been suggested to be an evolutionary conserved mechanism for non-shivering thermogenesis [82]. The amount of heat released by SERCA-catalysed ATP hydrolysis varies dependent on how much of the energy is used to pump Ca^2+^. For instance, sarcolipin enhances muscle thermogenesis by uncoupling SERCA-mediated ATP hydrolysis from Ca^2+^ transport [[83], [84], [85]] and SERCA2 has been implicated in ATP-dependent thermogenesis by Ca^2+^ cycling that can compensate for loss of UCP1 in beige adipose tissue [82]. The finding that the norepinephrine-induced increase in OCR was normal in SERCA2-deficient brown adipocytes (Suppl. Fig. 6C) is thus in line with that UCP1 is essential for norepinephrine-induced thermogenesis in BAT [86]. It is however possible that adrenergically stimulated thermogenesis is slightly blunted in SERCA2-deficient white adipocytes. Alternatively, the upregulation of UCP1 that we observe (Figure 6F) serves to compensate for the lack of this thermogenic mechanism in SERCA2-deficient adipocytes.

### 4.3 SERCA2 in fat accumulation

The lipodystrophic phenotype of adipocyte-specific SERCA2 knockout mice is in agreement with the proposed role of SERCA and Ca^2+^ homeostasis in fat storage [87]. However, our data suggest that fat storage and the capacity for *de novo* lipogenesis is normal in SERCA2 deficient adipocytes (Figure 6A). This proposes that SERCA2 is not essential for fat storage in mouse adipocytes, and that loss of SERCA2 rather makes the white adipocytes less viable, as supported by the increased number of crown-like structures, the increased Caspase 3 activation (Figure 3, Figure 4M) and the fewer, but larger, adipocytes under HFD-fed obese conditions (Figure 4K–L). Thus, both increased apoptosis and accelerated metabolism (as discussed above) provide plausible explanations for the reduced fat mass in these mice.

### 4.4 Conclusion

Collectively, our data emphasizes the importance of SERCA2 for preserved calcium homeostasis associated with proper ER and mitochondrial function in white and brown adipocytes. In obesity, reduction of adipocyte SERCA2 may thus contribute to adipose tissue dysfunction and the pathogenesis of metabolic disorders.

## Author contributions

IWA and CSO conceived the idea, supervised this work, interpreted data, and wrote the manuscript. They have contributed equally to this study and are shared senior authors. MBT and EB performed experiments and interpreted data, made figures, and wrote parts of the manuscript. BC, EP, YW, SM and CJ performed experiments and assisted in data interpretation. CJ and PS provided human adipocyte samples and clinical data. PR and PS interpreted data and gave valuable feedback to this work. All authors have approved the final version of this manuscript.
