# Supplementary material for: Adipocyte-specific ablation of the Ca2+ pump SERCA2 impairs whole-body metabolic function and reveals the diverse metabolic flexibility of white and brown adipose tissue
Source: Mol Metab. 2022 Jun 24;63:101535. doi: 10.1016/j.molmet.2022.101535 (PMC9287368; doi:10.1016/j.molmet.2022.101535)
Supplement: Multimedia component 1 [file mmc1.pdf]

Suppl. Fig. 1

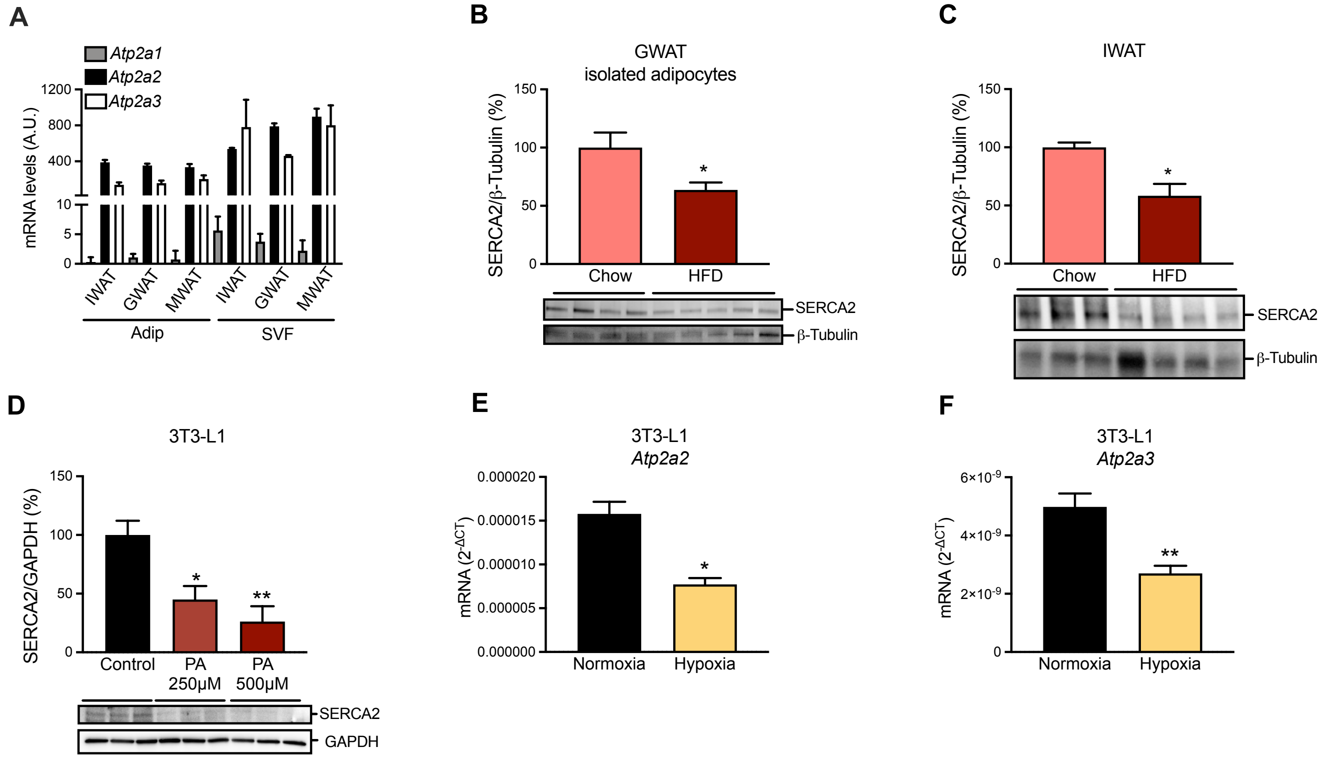

Supplemental figure 1. SERCA2 and 3 are the predominant SERCA paralogs in the adipose tissue. (**A**) *Atp2a1* (*Serca1)*, *Atp2a2* (Serca2) and *Atp2a3* (Serca*3*) gene expression in the adipocyte and the stromal vascular fractions (SVF) of inguinal (IWAT), gonadal (GWAT) and mesenteric white adipose tissue (MWAT) from chow-fed mice (N=4-6). (**B**) SERCA2 protein levels in isolated GWAT adipocytes from 16 week high fat diet (HFD)- and chow-fed wild type female mice (N=4-5). Chows are set to 100%. (**C**) SERCA2 protein levels in IWAT from 16 week HFD- and chow-fed wild type male mice (N=3-4). Chows are set to 100%. (**D**) SERCA2 protein levels in 3T3-L1 adipocytes treated with vehicle (BSA alone) or 250μM and 500μM palmitate conjugated with BSA (PA) (N=3/group). Controls are set to 100%. (**E-F**) *Serca2* (*Atp2a2*) and *Serca3* (*Atp2a3*) gene expression in 3T3-L1 adipocytes at normoxia vs. 24h hypoxia (N=6/group). All values are expressed as mean SEM; * $p<0.05$, ** $p<0.01$.

Suppl. Fig. 2

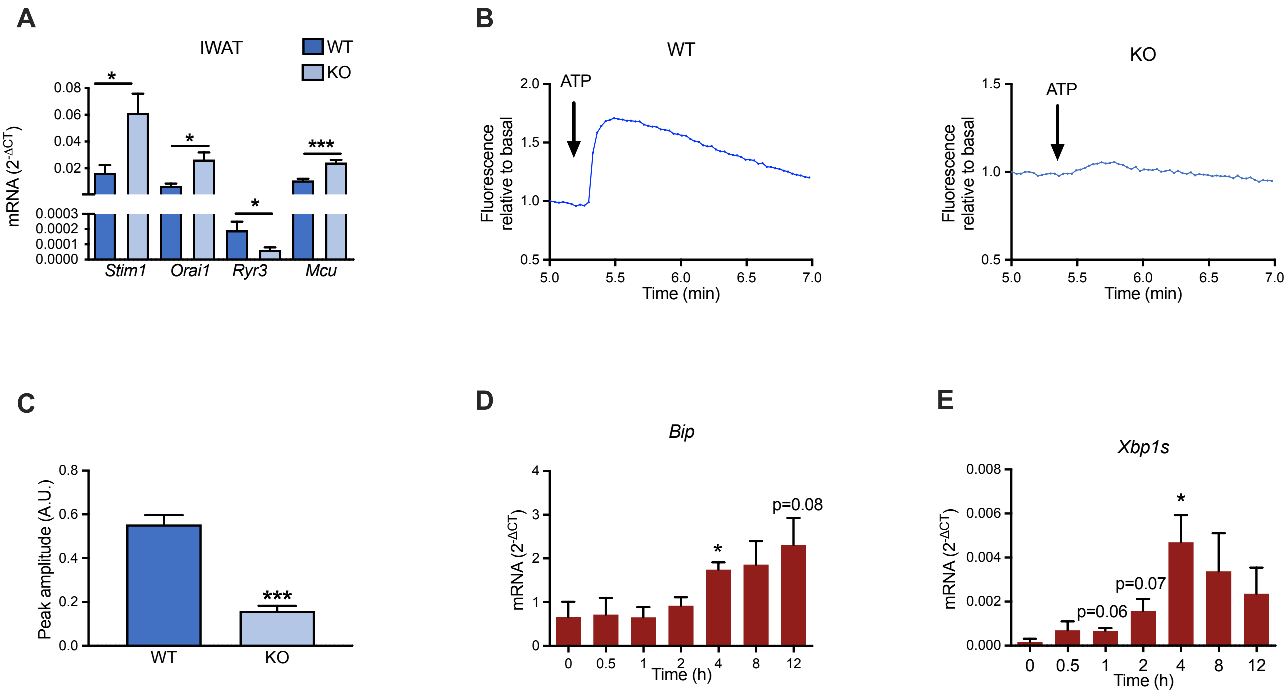

Supplemental figure 2. Inhibition of SERCA in adipocytes is associated with altered Ca^{2+} homeostasis and ER-stress. (**A**) Calcium transporter gene expression markers in IWAT in chow-fed Wildtype (WT) and adipocyte specific SERCA2 Knockout (KO) mice (N=6-10). (**B**) Example traces of mitochondrial Ca^{2+} and (**C**) ATP peak amplitude of the mitochondrial Ca^{2+} in response to ATP in differentiated WT and adipocyte-specific SERCA2 KO IWAT SVF adipocytes. *Bip* (**D**) and *Xbp1s* (**E**) mRNA levels in 3T3-L1 adipocytes in response to 20 μM CPA (*p<0.05 vs. time 0h). All values (N=3-6) are expressed as mean ± SEM; *p<0.05, ***p<0.001.

Suppl. Fig. 3

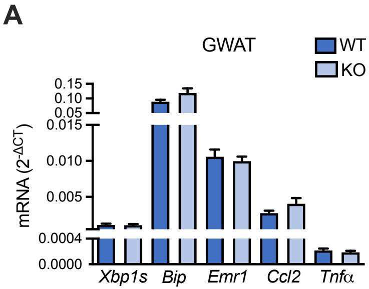

Supplemental figure 3. ER stress and inflammation markers are not altered in GWAT from adipocyte-specific SERCA2 knockout mice. ER stress and inflammation gene expression markers in GWAT in chow-fed WT and adipocyte-specific SERCA2 KO mice (N=6-10). All values are expressed as mean ± SEM.

Suppl. Fig. 4

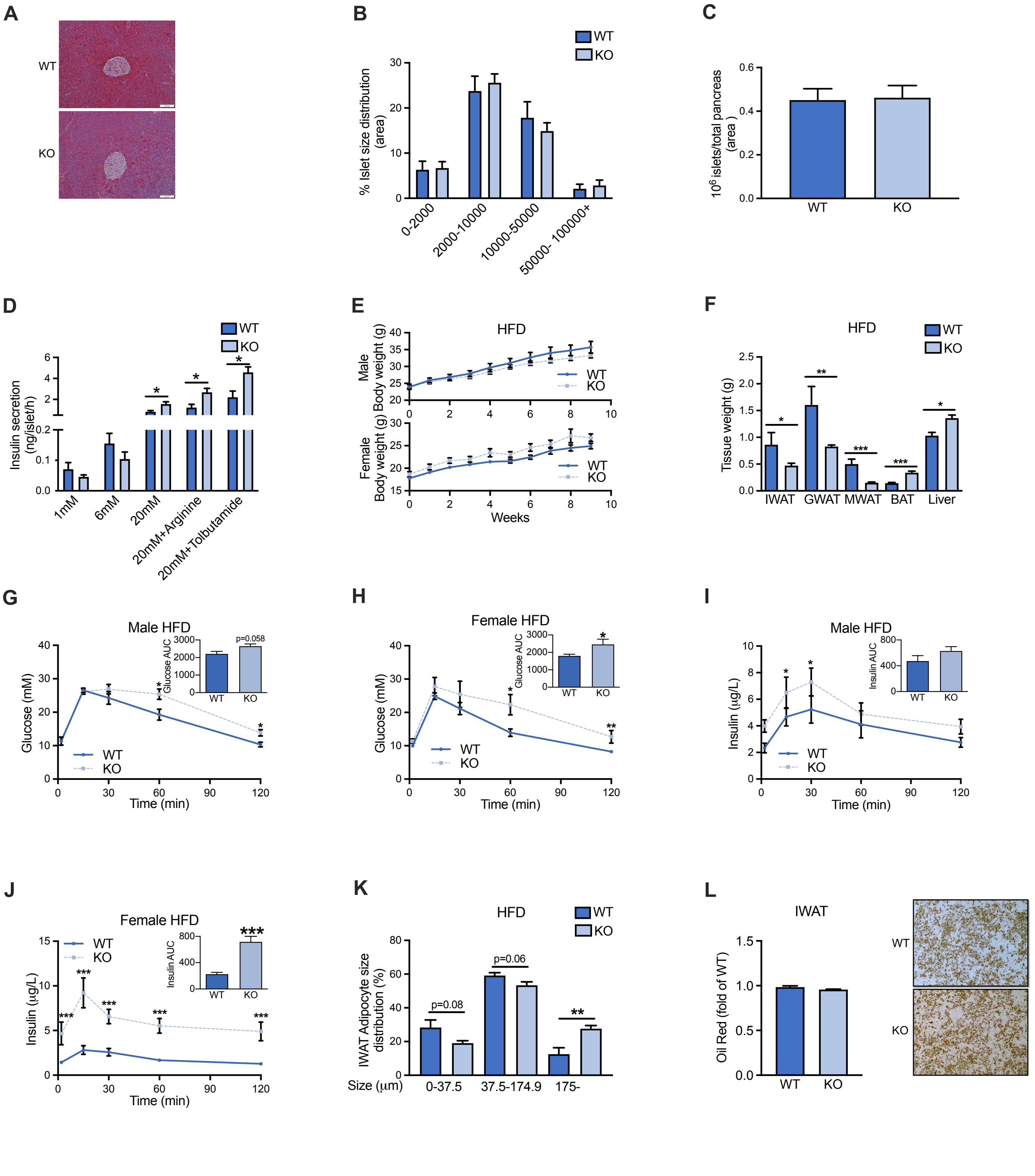

Supplemental figure 4. Adipocyte-specific SERCA2 knockout mice are glucose intolerant, but display increased glucose-stimulated insulin release. (**A**) Representative pancreas H&E sections, (**B**) % islets size distribution and (**C**) number of islets in pancreas sections from chow-fed WT and adipocyte-specific SERCA2 KO mice. (**D**) Insulin secretion from pancreatic islets isolated from chow-fed WT and adipocyte-specific SERCA2 KO mice (n=3 experiments; n=4 mice per genotype) in the presence of different concentration of glucose and Arginine or Tolbutamide, as indicated. (**E**) Body weight of male and female WT and adipocyte-specific SERCA2 KO mice during a 10-week high fat diet (HFD) time course. Male and female (**F**) IWAT, GWAT, MWAT, BAT and liver weight, (**G-H**) glucose and (**I-J**) insulin levels in response to an oral glucose load, and (**K**) IWAT adipocyte size distribution in 10-week-HFD-fed WT and adipocyte-specific SERCA2 KO mice. (**L**) Oil Red quantification and representative picture at day 8 of adipocytes differentiated from isolated WT and adipocyte-specific SERCA2 KO IWAT SVF. All values (N=6-10) are expressed as mean ± SEM; *p<0.05, **p<0.01, ***p<0.001 for WT vs KO.

Suppl. Fig. 5

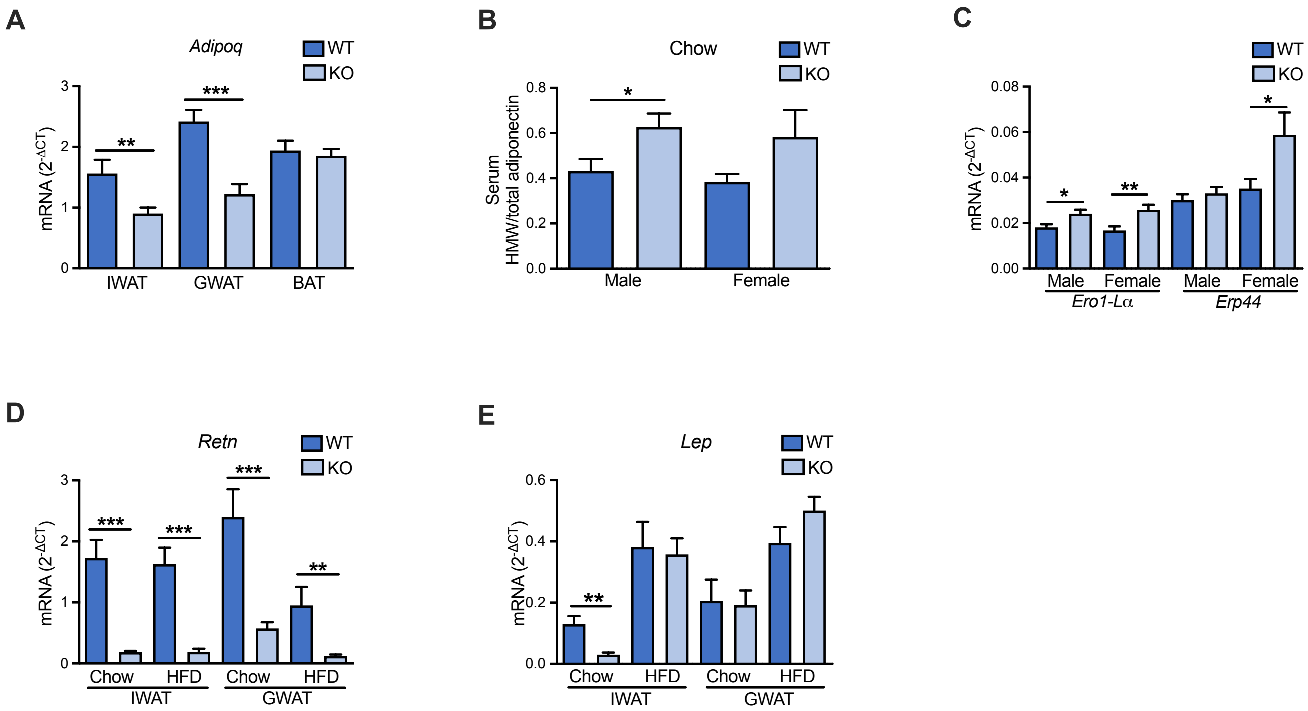

Supplemental figure 5. ER stress markers and adipocyte hormones are altered in adipocyte-specific SERCA2 knockout mice. (**A**) Adiponectin (*Adipoq*) expression in IWAT, GWAT and BAT in 10-week-HFD-fed WT and adipocyte-specific SERCA2 KO mice. (**B**) Serum HMW-Total adiponectin ratio in male and female chow-fed WT and adipocyte-specific SERCA2 KO mice. (**C**) *Ero1-Lα* and *Erp44* gene expression in male and female chow-fed WT and adipocyte-specific SERCA2 KO mice. IWAT and GWAT (**D**) resistin (*Retn*) and (**E**) leptin (*Lep*) gene expression in chow and 10-week-HFD-fed WT and adipocyte-specific SERCA2 KO mice. All values (N=6-10) are expressed as mean ± SEM; *p<0.05, **p<0.01, ***p<0.001 for WT vs KO.

Suppl. Fig. 6

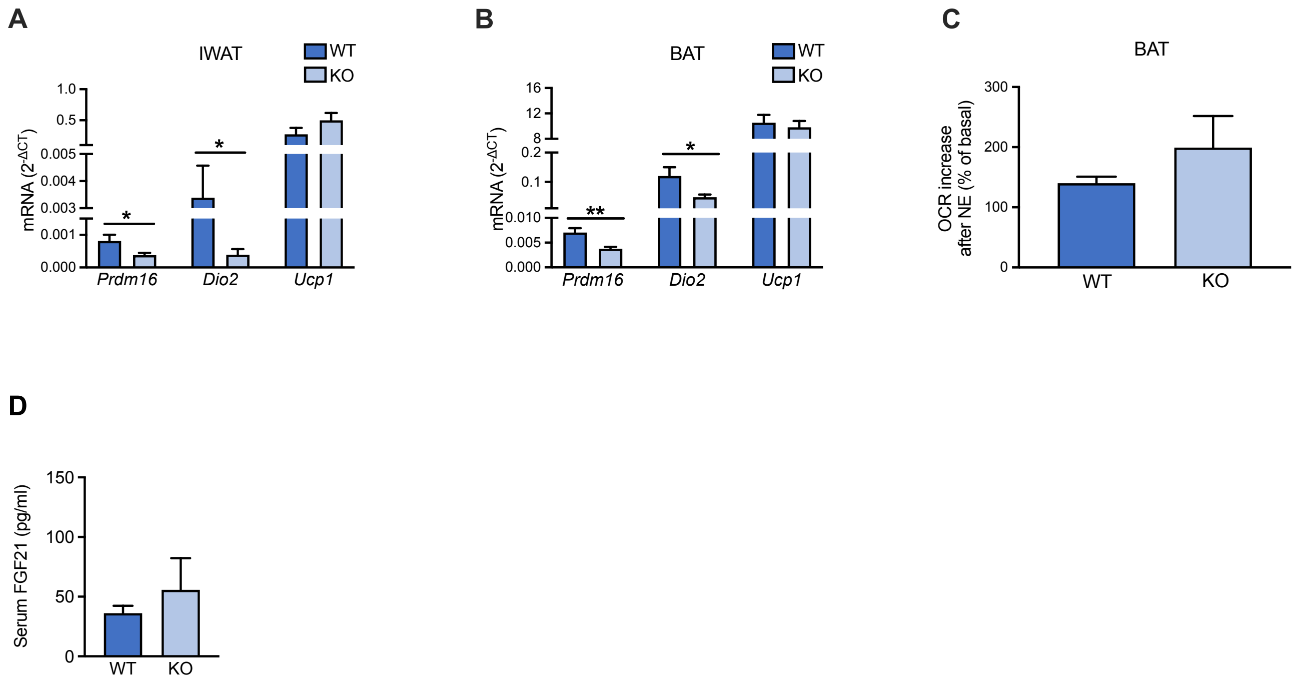

Supplemental figure 6. Effect of adipocyte-specific SERCA2 ablation on adipose tissue browning markers in white and brown adipose tissue. *Prdm16*, *Dio2* and *Ucp1* expression in (**A**) IWAT and (**B**) BAT in chow-fed WT and adipocyte-specific SERCA2 KO mice. (**C**) Norepinephrine (NE, 1μM)-induced increase in oxygen consumption rate (OCR) in brown adipocytes differentiated from BAT SVF of chow-fed WT and adipocyte-specific SERCA2 KO mice. (**D**) Serum *FGF21* levels in chow-fed WT and adipocyte-specific SERCA2 KO mice. All values (N=3-6) are expressed as mean ± SEM; *p<0.05, **p<0.01, ***p<0.001 for WT vs KO.
