# Supplementary material for: Adipocyte-specific ablation of the Ca2+ pump SERCA2 impairs whole-body metabolic function and reveals the diverse metabolic flexibility of white and brown adipose tissue
Source: Mol Metab. 2022 Jun 24;63:101535. doi: 10.1016/j.molmet.2022.101535 (PMC9287368; doi:10.1016/j.molmet.2022.101535)
Supplement: Multimedia component 2 [file mmc2.pdf]

Supplementary Table 1.

Gene	Forward primer (5'-3')	Reverse primer (5'-3')
Adipoq	TGTTCCTCTTAATCCTGCCCA	TGTTCCTCTTAATCCTGCCCA
Atp2a1 (Serca1)	TGTTTGTCCTATTTCGGGGTG	AATCCGCACAAGCAGGTCTTC
Atp2a2(Serca2)	GAGAACGCTCACACAAAGACC	CAATTCGTTGGAGCCCCAT
Atp2a3(Serca3)	CAATTCGTTGGAGCCCCAT	AAGAGGTCCTCAAACTGCTCC
Actb	GACCCAGATCATGTTTGAGA	GAGCATAGCCCTCGTAGAT
Bip	CCTCTCTGGTGATCAGGATA	CGTGGAGAAGATCTGAGACT
Emr1(F4/80)	CTTTGGCTATGGGCTTCCAGTC	GCAAGGAGGACAGAGTTTATCGTG
Fas	GGAGGTGGTGATAGCCGGTAT	TGGGTAATCCATAGAGCCCAG
Lep	GTCCAGGATGACACCAAAACC	GACAAACTCAGAATGGGGTGAA
Ccl2 (Mcp1)	ACTGAAGCCAGCTCTCTCTTCC	TTCCTTCTTGGGGTCAGCACAG
Tnfa	CAGGCGGTGCCTATGTCTC	CGATCACCCCGAAGTTCAGTAG
Ucp1	GTGAAGGTCAGAATGCAAGC	AGGGCCCCCTTCATGAGGTC
Retn	AGCGGATGAAGAACCTTTC	GGAGGAGACTGTCCAGCAAT
Xbp1	GGTCTGCTGAGTCCGCAGCAGG	GAAAGGGAGGCTGGTAAGGAAC
Erp44	TGCGGTCTTCCTGTCTTTAGC	AACGACACCAGTCAGCATAAAA
Ero1a	TTCTGCCAGGTTAGTGGTTACC	GTTTGACGGCACAGTCTCTTC
Dio2	TGTCTGGAACAGCTTCCTCC	CCATCAGCGGTCTTCTCCG
Prdm16	GCAGACCCTGTGGGAGTCCTGAAA	GCTCCCCTGTGTGTGTCCTCAGAT
Fgf21	ACCTGGAGATCAGGGAGGAT	CACCCAGGATTTGAATGACC
Casp3	ATGGAGAACAACAAAACCTCAGT	TTGCTCCCATGTATGGTCTTTAC
Stim1	TGACAGGGACTGTACTGAAGATG	TATGCCGAGTCAAGAGAGGAG
Orai1	GATCGGCCAGAGTTACTCCG	TGGGTAGTCATGGTCTGTGTC
Ryr3	ACCAGCAGGAGCAAGTACG	GGGGTCGTGTCAAAGTAGTCA
Mcu	CGCCAGGAATATGTTTATCCA	CTTGTAATGGGTCTCTCAGTCTCTT
